# Long-Acting Human PASylated Leptin Reaches the Murine Central Nervous System and Offers Potential for Optimized Replacement Therapy

**DOI:** 10.1021/acs.molpharmaceut.4c01503

**Published:** 2025-05-07

**Authors:** Volker Morath, Stefanie Maurer, Annette Feuchtinger, Rebecca Walser, Martin Schlapschy, Florian Bolze, Thomas Metzler, Johanna Bruder, Katja Steiger, Axel Walch, Martin Klingenspor, Arne Skerra

**Affiliations:** † Chair of Biological Chemistry, School of Life Sciences, Technical University of Munich, Freising 85354, Germany; ‡ Department of Nuclear Medicine, School of Medicine and Health, Technical University of Munich, Munich 81675, Germany; § Chair for Molecular Nutritional Medicine, School of Life Sciences, Technical University of Munich, Freising-Weihenstephan 85354, Germany; ∥ EKFZ—Else Kröner Fresenius Center for Nutritional Medicine, Technical University of Munich, Munich 81675, Germany; ⊥ Research Unit Analytical Pathology, 9150 Helmholtz Zentrum München, Neuherberg 85764, Germany; # Comparative Experimental Pathology (CEP), School of Medicine and Health, 9184 Technical University of Munich, Munich 81675, Germany

**Keywords:** adipokine, blood-brain barrier, light sheet fluorescence microscopy, obesity, PASylation, plasma half-life

## Abstract

Despite the multifaceted role of leptin for energy homeostasis and its broad therapeutic potential, the FDA/EMA-approved metreleptin constitutes the only leptin drug to date. To translate the promising results from previous studies on murine PASylated leptin with improved solubility and extended plasma half-life using PASylation technology—a biological alternative to PEGylation—we have developed a second-generation human leptin drug candidate and tested it rigorously *in vitro* and *in vivo*. To this end, the exposed hydrophobic Trp residue at position 100 in human leptin was replaced by Gln, which, together with the genetic fusion with a 600-residue PAS polypeptide, yielded a protein with high solubility, folding stability and receptor-stimulatory activity. In a pharmacokinetic (PK) study with wild-type mice, this modified human leptin showed an extended plasma half-life of 18.8 ± 3.6 h after subcutaneous (s.c.) injection. Furthermore, leptin-deficient mice were dosed s.c. with the modified human leptin carrying two different PAS fusion tags, PAS#1 or P/A#1, each comprising 600 residues. After only four doses, the disease phenotype, including morbid adiposity, hyperphagia, and hepatic steatosis, was completely reversed by both PASylated leptin versions, but not by the non-PASylated leptin if administered at the same dose. To assess its tissue distribution, P/A(200)-huLeptin^W100Q^ was doubly labeled with two fluorescent dyes, which were specifically attached to the leptin and the PAS moiety, respectively. Analysis of relevant mouse organs by light sheet fluorescence microscopy after clearance revealed colocalized signals in the kidney and liver, thus indicating general stability of the PAS-leptin fusion protein *in vivo*. However, discrete signals were observed in the hypothalamic region, only with leptin detectable in the choroid plexus, which implies cleavage of the PAS tag during transcytosis across the physiological barriers. This study should pave the way toward a second-generation leptin drug enabling prolonged dosing intervals.

## Graphical abstract

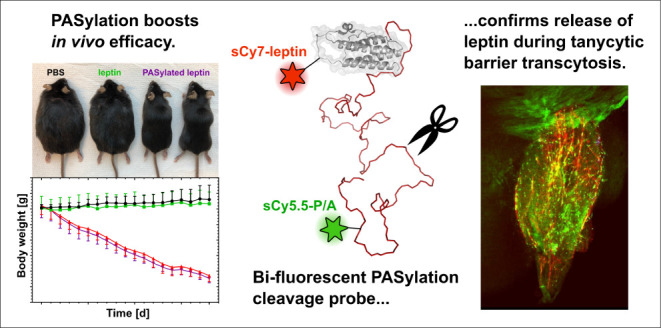

## 1 Introduction

Leptin is a physiologically crucial adipokine that is secreted by adipocytes into the bloodstream in proportion to the adipose tissue mass and, thus, communicates the state of energy storage to the brain.^1–3^ In this regard, leptin plays a central role in various processes such as feeding behavior and energy expenditure as well as immune and reproductive functions.^4^ From a biochemical point of view, mature leptin (UniProt ID: P41159) is a small, unglycosylated protein hormone with 146 amino acid residues (16 kDa). Its fold comprises four α-helices, which are structurally stabilized by a single disulfide bridge.^5^

Leptin undergoes active transport from the blood into the brain by a saturable system located at the vascular blood-brain barrier (BBB), the choroid plexus (CP) and the tanycytic barrier.^6^ After that, leptin is sensed by the neuronal leptin receptor, LepR also known as ObR, in the arcuate nucleus at the base of the hypothalamus. LepR exists as a long isoform capable of JAK-STAT signaling in the neurons (LepRb) and as shorter isoforms (in particular, LepRa) that lack signaling activity but may function as transporter across the vascular barrier.^6–8^ Apart from the hypothalamus, LepRb is expressed in the CP, the third ventricle, the median eminence (ME) as well as the brain vasculature.^9,10^

To execute its effects in the central nervous system (CNS), leptin needs to traverse one of three saturable barriers: (i) the vascular BBB formed by endothelial cells of the capillary wall, (ii) the blood-cerebrospinal fluid barrier (BCSFB), located in the CP, or (iii) the tanycytic barrier at the circumventricular organs.^8,11^ While paracellular transport is largely prevented by tight junctions, leptin transport across these barriers occurs via receptor-mediated transcytosis and is saturated at blood leptin levels of 50 ng/mL (∼3 pmol/mL).^6^ Notably, leptin replacement therapy often uses suprapharmacological doses as high as 1000 ng/mL (∼60 pmol/mL) or above.^6^

Shortly after the discovery of leptin in 1994,^12^ its central regulatory effects on energy balance were described. Initially, high expectations were raised with regard to a potential cure for obesity, as well as its comorbidities, based on the observation that leptin can induce strong body weight loss in both healthy and leptin-deficient obese (*Lep^ob/ob^*) mice.^13^ However, leptin could not keep up with these promises as the impressive weight-lowering effect seen in rodents was not confirmed in human trials.^14^ In fact, obese human patients often do not suffer from a lack of leptin but from reduced sensitivity toward this adipokine.^13^ This phenotype, generally known as ″leptin resistance″, limits the applicability of leptin therapy to certain metabolic disorders, for example congenital leptin deficiency and different forms of lipodystrophy.^15^ Several mechanisms of leptin resistance have been discussed, including obesity-associated hyperleptinemia, silenced leptin receptor signaling as well as impaired access of leptin to the CNS.^15,16^ Due to this kind of reduced leptin responsiveness, even a PEGylated leptin drug candidate with extended plasma half-life did not provoke weight loss in obese patients under caloric restriction.^17^ Nevertheless, leptin replacement therapy with unmodified recombinant leptin, metreleptin (Myalept), is well established for patients suffering from congenital leptin deficiency.^13,18^

Early studies on the synthesis rate and clearance of endogenous leptin in lean and obese men indicated a very short plasma half-life of only 25 ± 4 min.^2^ Thus, the therapeutic use of metreleptin relies on daily subcutaneous (s.c.) administration, which leads to retarded distribution into the blood—as well as better convenience for patients—compared to intravenous (i.v.) dosing. Currently, only Myalept is approved for leptin replacement therapy by the U.S. Food & Drug Administration (FDA) and the European Medicines Agency (EMA). Metreleptin is a recombinant protein produced in E. coli comprising the mature human leptin, lacking its native signal sequence and carrying an additional N-terminal start Met-residue instead.^13^ Metreleptin is administered using insulin needles, but if the dosing requires a volume larger than 0.8 mL even two injections per day are common.^9^

Considering that an effective leptin replacement therapy requires such frequent dosing over an extended period, we have previously applied PASylation technology^19^ to develop murine leptin variants with a prolonged plasma half-life, thus boosting the pharmacodynamic (PD) effect while drastically reducing the dosing frequency in mice.^20^ PASylation involves the fusion of a pharmacologically active protein or peptide with a strongly hydrophilic polypeptide comprising only the small natural L-amino acids Pro, Ala, and, optionally, Ser (PAS-polypeptide). The conformationally disordered structure, with expanded hydrodynamic volume, of this PEG-like biopolymer leads to an increased molecular size of the PASylated leptin and, consequently, to retarded kidney filtration.^19,21^ A first proof-of-principle study with wild-type C57BL/6J mice^20^ was followed by the successful treatment of genetically leptin-deficient *Lep^ob/ob^* mice^22^ as well as an application in a mouse model of lipodystrophy.^23^ Furthermore, the PASylated murine leptin was used in an investigation on the reversal of leptin resistance in POMC neurons.^24^

With the goal of translating these promising findings to the human situation, an amino acid sequence alignment between murine and human leptin revealed high homology (84.9% identical residues for the mature protein). However, a solvent-exposed Trp residue occurs specifically in the Hominoidea at position 100, whereas essentially all other taxa exhibit a more hydrophilic side chain at this position (Figure S1). Already during the initial X-ray structural analysis of human leptin, the strongly hydrophobic Trp side chain appeared to cause aggregation, which was overcome by its replacement with the much more polar residue Glu (mutant W100E).^5^ Further research at Eli Lilly^25^ and at Amylin/AstraZeneca^26^ focused on leptin mutants with preserved receptor activity but increased stability and solubility. In fact, limited solubility was described for human leptin at neutral pH, with 2–3 mg/mL versus 43 mg/mL for the murine protein,^27^ thus posing a severe caveat for effective leptin therapy with an intended s.c. dose of up to 10 mg/day.^13^ Indeed, the observed prevalence of anti-leptin antibodies in >95% of the patients during clinical trials may be caused by protein aggregation.^13,28,29^ Therefore, the FDA initiated the so-called Myalept Injection Risk Evaluation and Mitigation Strategy (REMS).^9^ Consequently, improving the solubility of functional human leptin, both by removal of the hydrophobic Trp side chain and fusion with a new version of the hydrophilic PAS biopolymer, which also effects strongly prolonged plasma half-life, should offer new prospects for safety and tolerability upon therapeutic application.

## 2 Materials and Methods

### 2.1 Bacterial Production and Purification of Leptin

The bacterial expression plasmid pASK37 was used to produce the different recombinant leptin variants under transcriptional control of the *lac*UV5 promoter/operator.^30^ The coding region for human leptin was obtained by gene synthesis (GeneArt/Thermo Fisher Scientific, Sankt Leon-Rot, Germany), and mutations were generated by QuikChange site-directed mutagenesis (Agilent, Santa Clara, CA).

The open reading frames of the mature leptin variants intended for comparative biochemical analyses were preceded by an N-terminal Met-Pro-His_6_-Ala-Ser_3_-Ala extension to allow efficient cytoplasmic biosynthesis and facilitate purification from the whole cell extract via immobilized*-*metal ion affinity chromatography (IMAC). The PAS gene sequences were inserted at the ultimate Ala residue within this leader sequence using a singular *Sap*I restriction site as previously described.^19^ E. coli Origami B^31^ was transformed with the corresponding expression plasmid and cultivated in 2 L terrific broth (TB) medium (12 g/L bacto tryptone, 24 g/L yeast extract, 0.4% (v/v) glycerol, 72 mM K_2_HPO_4_, 17 mM KH_2_PO_4_) containing 100 mg/mL ampicillin (Amp) at 30 °C in a baffled 5 L Erlenmeyer flask at 110 rounds per minute (rpm). At OD_550_ ≈ 1, recombinant gene expression was induced by adding isopropyl-β-D-thiogalactopyranoside (IPTG) to a concentration of 0.5 mM, and incubation was continued for 18 h. While the PAS(200) fusion protein was obtained in a soluble state after bacterial lysis in a French pressure cell (SLM Aminco, Urbana, IL), the unmodified recombinant leptin formed inclusion bodies under the same conditions of gene expression. These inclusion bodies were washed twice with phosphate-buffered saline (PBS; 4 mM KH_2_PO_4_, 16 mM Na_2_HPO_4_, 115 mM NaCl, pH 7.4), then solubilized in 6 M guanidinium chloride, 10 mM β-mercaptoethanol, 10 mM EDTA pH 8.0, and refolded by dropwise dilution into SA buffer (100 mM Tris/HCl, 50 mM NaCl, 1 mM EDTA, pH 8.0). Subsequent protein purification was accomplished using (NH_4_)_2_SO_4_ precipitation, in case of the PASylated variants, as well as IMAC and a final size exclusion chromatography (SEC) in PBS following published procedures.^20^ Leptin fusion proteins with 200 PAS residues were used for the comparative biophysical characterization due to their more facile protein purification.

Longer acting leptin versions intended for *in vivo* studies were equipped with a PAS-tag comprising 600 residues utilizing either the PAS#1^19^ or the P/A#1 sequence.^32^ These PAS-leptin fusion proteins were also produced in the cytoplasm of E. coli Origami B with a few modifications. First, a seamless cloning cassette featuring two oppositely arranged type IIS *Sap*I restriction sites was applied instead of the N-terminal Ser_3_Ala linker, and the His_6_-tag was omitted. In this case, protein purification was achieved by initial precipitation with 1 M (NH_4_)_2_SO_4_ (see Figure S5), which was followed by anion exchange chromatography (AEX) on a Resource Q column, using 20 mM Tris/HCl pH 8.5 as running buffer and an ascending NaCl concentration gradient, as well as final SEC on a Superdex 200 column (both GE Healthcare/Cytiva, Munich, Germany) using PBS as the mobile phase.

Protein concentrations were determined by UV absorbance measurements at 280 nm using a calculated absorption coefficient^33^ of 8,605 M^–1^·cm^–1^ (note that the PAS sequence is devoid of aromatic side chains and does not contribute to UV absorption at this wavelength). Protein samples on Coomassie-stained SDS-PAGE were quantified via fluorescence intensity^34^ using an Odyssey scanner (LI-COR Biosciences, Lincoln, NE) in the 700 nm fluorescence channel. For proteins applied *in vivo*, the endotoxin content was confirmed to be <10 EU/mL using the Endosafe-PTS system (Charles River Laboratories, Wilmington, MA). Purified proteins were snap-frozen in liquid nitrogen until use. Electrospray ionization mass spectrometry (ESI-MS) was performed on maXis and impact II Q-TOF instruments (both from Bruker Daltonics, Bremen, Germany) in the positive ion mode for P/A(600)-huLeptin^W100Q^, revealing a mass of 64,250.5 Da (calculated: 64,249.8 Da), thus indicating the complete processing of the N-terminal Met residue, oxidative formation of the unique disulfide bridge and absence of post-translational modifications.

### 2.2 Circular Dichroism (CD) Spectroscopy

Differences in folding stability were assessed by thermal unfolding using circular dichroism (CD) spectroscopy on a J-810 spectropolarimeter (Jasco, Pfungstadt, Germany) equipped with a PT-423S Peltier element.^35^ Purified leptin variants were dialyzed against 20 mM K–P_i_, 50 mM K_2_SO_4_, pH 7.5, and measured at a concentration of 2 μM in a quartz cuvette. Thermal protein unfolding was monitored at a wavelength of 222 nm (negative band maximum for α-helical proteins) by heating from 20 to 95 °C at a rate of 60 K/h. Data were analyzed with the CDpal (ver. 2.18) software^36^ using its Autofit function for N → D thermal denaturation, yielding the melting temperature (*T*_m_) for each leptin variant.

### 2.3 Biomolecular Interaction Analysis

Real-time surface plasmon resonance (SPR) interaction analyses between leptin variants produced in this study and a recombinant LepR-Fc fusion protein (389-LR-100/CF; R&D Systems, Minneapolis, MN)^37^ were performed on a BIAcore 2000 system (BIAcore, Uppsala, Sweden) at 25 °C using HBS-T (20 mM HEPES/NaOH pH 7.5, 150 mM NaCl, 0.05% (v/v) Tween-20) as running buffer. LepR-Fc was immobilized on a CM5 sensorchip (BIAcore) using an amino coupling kit (BIAcore) and 10 mM Na-acetate, 10 mM NaCl, pH 5.0 as immobilization buffer, resulting in ΔRU ≈ 925. Purified His_6_-PAS­(200)-huLeptin^W100Q^, His_6_-PAS­(200)-huLeptin^W100E^ and His_6_-PAS­(200)-muLeptin were each diluted to 32 nM in running buffer and, starting from that, a 1:2 dilution series was prepared. These analyte solutions were each applied for 240 s, whereas dissociation was subsequently followed for 1,800 s at a continuous flow rate of 25 μL/min. The kinetic parameters were determined by data fitting to a Langmuir binding model for bimolecular complex formation using BIAevaluation software (ver. 4.1; BIAcore). The resulting multicycle sensorgrams were corrected by double subtraction of the corresponding signals measured for the in-line control blank channel and an averaged baseline determined from several buffer blank injections.

### 2.4 Dual Luciferase Assay

The potencies of recombinant leptin versions in cell culture were assessed as previously described.^20^ In brief, HEK293 cells were transiently transfected using the calcium phosphate method with three different expression vectors: (i) human LepRb-pDEST26 (Source BioScience, Nottingham, UK); (ii) pAH32 (STAT3-responsive Photinus luciferase reporter gene; kindly provided by Dr. C. Bjorbaek); (iii) phRG-b (constitutive Renilla luciferase expression vector; Promega, Mannheim, Germany). The different recombinant leptin versions were added to the culture medium at rising concentrations (50 fM to 500 nM), followed by incubation of the transfected cells for 18 h. Luciferase activities were assessed in a Sirius luminometer (Berthold Technologies, Bad Wildbad, Germany), plotted against the applied leptin concentrations (four outlier values were excluded, one each for huLeptin^W100^, huLeptin^W100Q^, two for PAS(200)-muLeptin), and fitted according to a sigmoidal dose–response curve with variable slope using Prism 9 (GraphPad Software, Boston, MA) to calculate the half-maximal effective concentrations (EC_50_).

### 2.5 Animal Experiments

Animal experiments were conducted with permission from the district government of Upper Bavaria (license numbers: 55.2.1.54-2532-183-11, 55.2.1.54-2532-216-15, and 55.2-2532.Vet_02-18-128) and in accordance with the German animal welfare law. During the experiments, mice were single-housed in individually ventilated cages (Tecniplast, Hohenpeißenberg, Germany) in a specified pathogen-free (SPF) animal facility at 22 °C under a relative humidity of 55% and controlled light/dark (12 h/12 h) conditions. Mice had *ad libitum* access to standard rodent chow diet (V1124-300; Ssniff Spezialdiäten, Soest, Germany) and water.

### 2.6 Pharmacokinetic (PK) Study in Wild-Type Mice

10–12 weeks-old male C57BL/6J mice, with an average body weight (b.w.) of 24 g, were injected s.c. with a solution of purified P/A(600)-huLeptin^W100Q^ in PBS at a dose of 287 pmol/g b.w. Blood samples were collected from three groups (I, II, III; *N* = 3 per group) as follows: (group I) 10 min, 2 h, 6 h, 24 h; (group II) 30 min, 3 h, 36 h, 48 h; (group III) 1 h, 4 h, 8 h, 12 h. From each blood sample, the plasma was prepared by centrifugation in a benchtop centrifuge at 1500 ×*g* and 4 °C for 5 min and stored at −20 °C. To determine the plasma half-life in mice, the concentration values, c­(t), were determined for each time point from ELISA measurements of these samples (see below) and plotted against the time post injection, t. The data were numerically fitted using Phoenix WinNonlin 6.3 software (Pharsight, St Louis, MO), assuming a first-order invasion from the s.c. tissue and first order-elimination by the kidneys as described by the Bateman function:

$$c\left(t\right)=\frac{D\times k_{01}}{V\times \left(k_{01}-k_{10}\right)}e^{-k_{10}\times t}-e^{-k_{01}\times t}$$

wherein D is the applied dose in mg·kg^–1^ b.w., V is the volume of distribution in ml·kg^–1^ b.w., *k*_01_ is the absorption rate and *k*_10_ is the elimination rate, both in h^–1^. The plasma half-life of P/A(600)-huLeptin^W100Q^ was determined from this fit.

### 2.7 ELISA to Quantify PASylated Leptin in Plasma Samples

For the quantification of P/A(600)-huLeptin^W100Q^ in an enzyme-linked immunosorbent assay (ELISA), a 96-well microtiter plate (NUNC Maxisorb, Thermo Fisher Scientific) was coated with 50 μL 20 μg/mL Avi-PA­(S) MAb2.1 antibody (XL-protein, Freising, Germany) in PBS per well for 2 h. After removal of the coating solution, the wells were blocked with 200 μL 3% (w/v) bovine serum albumin (BSA) in PBS/T (PBS supplemented with 0.1% (v/v) Tween-20) for 1 h and washed three times with PBS/T. The plasma samples from mice were applied in dilutions of 1:1,600 in PBS/T containing 0.0625% (v/v) mouse plasma from an untreated animal and incubated for 1 h. The wells were then washed three times with PBS/T and incubated for 1 h with 50 μL of a 1:5,000 diluted solution of Avi-PA­(S) MAb1.1-alkaline phosphatase (AP) conjugate (0.8 mg/mL; XL-protein). After washing twice with PBS/T and twice with PBS, the chromogenic reaction was started by adding 50 μL of 0.5 mg/mL *p*-nitrophenyl phosphate in 100 mM Tris/HCl pH 8.8, 100 mM NaCl, 5 mM MgCl_2_. After incubation for 10 min at 30 °C, the absorbance at 405 nm was measured using a Synergy 2 plate reader (BioTek Instruments, Bad Friedrichshall, Germany). Concentrations of P/A(600)-huLeptin^W100Q^ in the initial plasma samples were determined by comparison with standard curves from dilution series of the purified recombinant protein in PBS/T containing 0.0625% (v/v) untreated mouse plasma, also taking into consideration the applied dilution factor.

### 2.8 PK Modeling

PK was modeled based on differential equations using the Berkeley Madonna software (ver. 8.3.18; Berkeley Madonna, Albany, CA). A one-compartment model with first-order absorption from the dosing site^38^ was adapted to a repeated dose study. The transport coefficient (*k*_a_) from the peripheral dosing compartment (lep_per) to the central blood compartment (lep) and the clearance value (Cl600) were derived from a curve fit to experimental data from the s.c. PK study of P/A(600)-huLeptin^W100Q^ in wild-type mice described above. Of note, *Lep* ^*ob/ob*^ mice are 79% heavier than age-matched wild-type mice and have 21% larger blood volume.^39^ Using this correction, the normal blood content of 6% (mL/g b.w.) for rodents^40^ was used to estimate a plasma volume (V) of ∼2.0 mL for the *Lep^ob/ob^* mice. For the simulation of the unmodified leptin (huLeptin^W100Q^), the published clearance parameters for the elimination of recombinant muLeptin (CL_0_)^20^ were used, whereas it was assumed that *k*_a_ was the same as measured here for P/A(600)-huLeptin^W100Q^. The change in b.w. upon treatment of the *Lep^ob/ob^* mice was anticipated from data obtained from the previous study on muLeptin in the same mouse strain^23^ and implemented as a linear decrease. The physiological leptin level in wild-type C57BL/6J mice at 2 months age is known to be ∼5 ng/mL,^41,42^ which corresponds to ∼0.3 pmol/mL and can serve as a reference for interpretation of the plasma leptin levels obtained from this simulation. For details of the mathematical PK model, see the Supporting Information.

### 2.9 PD Study in *Lep* ^*ob/ob*^ Mice

Obese, homozygous leptin-deficient (*Lep* ^*ob/ob*^) mice were obtained from our own B6.Cg-*Lep* ^*ob*^/J breeding colony (JAX Lab stock #000632) by mating heterozygous male *Lep* ^*+/ob*^ with heterozygous female *Lep* ^*+/ob*^ mice. Male *Lep* ^*ob/ob*^ mice with an initial b.w. of 45.7 ± 2.7 g at the age of 10–11 weeks were equally distributed into four cohorts with four animals each. B.w., body temperature and food intake were recorded for 19 days (starting on day 0). Body composition was determined via nuclear magnetic resonance (NMR) using an LF50H TD NMR Analyzer (Bruker, Billerica, MA), once at the beginning of the experiment (day 0) and on day 17, then together with oral glucose tolerance testing (oGTT, see below). At days 0, 5, 10, and 15 of the study, mice received an s.c. injection of each human leptin variant, huLeptin^W100Q^, PAS(600)-huLeptin^W100Q^ or P/A(600)-huLeptin^W100Q^ (100 pmol/g b.w.), or PBS (2.65 μL/g b.w.) as negative control. Mice were euthanized on day 18 via CO_2_ exposure.

### 2.10 Oral Glucose Tolerance and Body Composition

oGTT was performed after a 6 h fasting period during the light phase (8 a.m. to 2 p.m.), along with the quantification of the body composition via NMR. To account for the fact that the specific metabolic activity of fat is only 20% compared with lean tissue, the obese mice received a single oral glucose load at a dose of 2.7 mg/(g lean mass +0.2 × g fat mass). Blood was sampled from the tail tip through a small incision. Glucose concentrations were quantified with a hand-held Freestyle Lite glucometer (Abott, Wiesbaden, Germany) before and after gavage. The total area under the curve (AUC) was calculated using the trapezoid method.^43^

### 2.11 Histological Assessment of Hepatic Steatosis

Liver samples were fixed in 4% (w/v) formalin solution for up to 7 days, dehydrated and embedded in paraffin. Serial 2 μm sections were prepared with a HM355S rotary microtome (Thermo Fisher Scientific). Hematoxylin and eosin (H&E) staining was performed on deparaffinized sections with Eosin and Mayer’s Haemalaun (Morphisto, Frankfurt am Main, Germany). Staining of glycogen storage was performed using periodic acid and Schiff’s reagent (both from Carl Roth, Karlsruhe, Germany) and Mayer’s Haemalaun (Waldeck, Münster, Germany) as counter stain. Representative images were collected on an Aperio AT2 digital pathology slide scanner using ImageScope ver. 12.3 software (both from Leica Biosystems, Wetzlar, Germany). A ’blinded’ expert pathologist evaluated the grade of hepatic steatosis using a scoring system ranging from 0 (normal) to 3 (severe abnormalities).^44^

### 2.12 Preparation of a Fluorescently Labeled PAS-Leptin Protein

For the preparation of the doubly fluorescence-labeled PASylated leptin used in imaging experiments, a ketone-reactive and an amine-reactive fluorescent dye with distinct excitation wavelengths were employed. A one-plasmid expression system was used to site-specifically incorporate the non-canonical amino acid *p*-acetyl-l-phenylalanine (Apa) via amber suppression in E. coli.^45^ The N-terminus of the coding region for huLeptin^W100Q^ was extended by the Met-Pro-His_6_-Gly-Apa-Ser_3_-Ala sequence (wherein Apa was encoded by the amber stop codon) via a pair of hybridized oligodeoxynucleotides that were inserted between the *Nde*I and *Sap*I restriction sites. Subsequently, the entire coding region was cloned on the plasmid pSB8.12e2^45^ via the *Xba*I and *Hin*dIII restriction sites and, after that, a P/A(200) gene cassette was inserted into the *Sap*I restriction site as described above. The resulting expression plasmid, pSB8.12e2-Apa-P/A(200)-huLeptin^W100Q^, was used to transform E. coli Origami B. Preparative protein production was carried out in six 2 L cultures with TB/Amp medium. Bacteria were cultivated at 30 °C and 120 rpm until OD_550_ ≈ 0.5 was reached, upon which expression of the engineered aminoacyl-tRNA synthetase was induced with 50 ng/mL anhydrotetracycline (aTc). After 30 min, the medium was supplemented with 1 mM L-Apa, and gene expression for the PASylated leptin was induced by adding 0.5 mM IPTG.^45^

After incubation overnight (12 h), the cultures were harvested by centrifugation, and cell disruption was carried out using a PandaPLUS 1000 homogenizer (GEA, Düsseldorf, Germany). The bacterial cell extract was dialyzed against SA buffer and subjected to (NH_4_)_2_SO_4_ precipitation as described above. The precipitate was redissolved in 20 mM Tris/HCl pH 8.0, 500 mM NaCl and purified on a Ni^2+^-charged HiTrap IMAC HP column (Cytiva), yielding essentially pure protein with a total yield of 18.4 mg. The incorporation of the non-canonical Apa residue was confirmed by ESI-MS, revealing a predominant species at 33,502.83 Da (calculated mass: 33,503.30 Da). Again, the start Met residue of the fusion protein had been processed during the cytoplasmic production, resulting in a Pro imino acid residue at its N-terminus.

To compare the conjugation via the Apa side chain with aminooxy- and hydrazide-functionalized fluorescent dyes at analytical scale, aminooxy-sulfoCyanine5 (sCy5 *alias* Cyanine 647; Biotium, Fremont, CA) and hydrazide-sulfoCyanine5.5 (sCy5.5; AAT Bioquest, Sunnyvale, CA) were used for coupling in Na-acetate pH 4–5.5 for 24 h at 4 °C. Resulting protein conjugates were purified by SEC and analyzed with regard to chemical stability and also for the possibility to isolate the reaction product by AEX chromatography (see Figure S8). Based on these findings, hydrazide-sCy5.5 was selected to prepare the PAS-leptin conjugate for subsequent *in vivo* studies. To this end, the purified protein (∼3 mL at ∼3 mg/mL) was dialyzed against 50 mM Na-acetate pH 5.0, 150 mM NaCl, 10% (v/v) glycerol. To avoid protein aggregation by the addition of an organic cosolvent, 1 mg of the highly water-soluble hydrazide-sCy5.5 was directly added as a solid, followed by incubation at 4 °C for 4 days. AEX on a Resource Q column was used to isolate the sCy5.5-Apa-P/A(200)-huLeptin^W100Q^ conjugate, using the absorption of the fluorescent dye (ε_683_ = 250,000 cm^–1^·M^–1^) to determine its concentration.

After dialysis against 100 mM Na-carbonate pH 8.6, a 4-fold molar amount of NHS-sulfoCyanine7 (sCy7; Lumiprobe, Hannover, Germany) was added, followed by incubation overnight at 4 °C. Finally, the doubly labeled PASylated leptin was subjected to SEC on a Superdex S200 10/300 HR column using PBS as running buffer. Fluorescent dyes were quantified by absorbance measurements at 683 and 750 nm (sCy7: ε_750_ = 240,600 cm^–1^·M^–1^), respectively, using an Ultrospec 2100 pro spectrophotometer (GE Healthcare). Fluorescence of labeled protein samples after separation by SDS-PAGE was probed using an Odyssey scanner via detection of sCy5.5 in the 700 nm channel. Alternatively, sCy5 was detected using an Ettan DIGE scanner (GE Healthcare).

### 2.13 *In Vivo* Metabolization Study and Light Sheet Fluorescence Microscopy (LSFM)

Female C57BL/6J mice were purchased from Charles River Laboratories (Wilmington, MA). For the imaging experiment, 300 pmol/g b.w. (∼7.5 nmol ≈ 250 μg per mouse) of the sCy5.5-PAS-leptin-sCy7 double conjugate was injected i.v. and the mice were housed for 8 h to allow distribution and metabolization of the molecular probe. Fifteen min ahead of the planned end of the experiment, mice received an i.p. injection of 3 nmol *Bandeiraea simplicifolia* lectin (Sigma-Aldrich, St. Louis, MO) which had been conjugated with Dy-594 NHS ester (Dyomics, Jena, Germany). Transcranial perfusion was carried out for 15 min at a flow rate of ∼4 mL/min with cold PBS, and subsequently for 10 min with PAXgene Tissue Stabilizer (Qiagen, Hilden, Germany). Finally, the brain, liver, kidneys, lungs, spleen, white and brown adipose tissue were excised and fixed for 24 h.

Tissue clearance of PAXgene-fixed organs was performed as previously described^46^ using a chemical procedure with different organic solvents according to the 3DISCO standard protocol.^47^ The cleared whole organs were imaged on a light sheet fluorescence microscope (UltraMicroscope II; LaVision BioTec, Bielefeld, Germany). Fluorescence signals arising from the Dy-594-lectin were detected using a band-pass filter (excitation: 576/23 nm; emission: 620/31 nm). The two fluorescent dyes incorporated into the P/A(200)-huLeptin^W100Q^ imaging probe were detected in a distinct manner using appropriate filters for sCy5.5 (excitation: 680/30 nm; emission: 722/30 nm) and sCy7 (excitation: 740/35 nm; emission: 795/50 nm). 3D image reconstruction and rendering were performed using arivis Vision4D software (ver. 3.0; Carl Zeiss, Oberkochen, Germany).

### 2.14 Statistics

*In vivo* data on food intake, b.w., oGTT and body composition were analyzed for statistical significance using one-way ANOVA with Tukey multiple comparison test using Prism 9 software. Mean values are reported with error bars indicating standard deviations. Significance levels were annotated as follows: not significant (ns) *p* > 0.05; *p ≤ 0.05; **p ≤ 0.01; ***p ≤ 0.001; ****p ≤ 0.0001.

## 3 Results

### 3.1 Potency and Folding Stability of Leptin Orthologs

Our first step toward a leptin drug candidate with extended plasma half-life was the switch from the previously characterized murine PASylated leptin^20^ to its human counterpart. However, in this endeavor, the well-known aggregation tendency of natural human leptin constituted a problem.^27^ Thus, we compared the murine and human leptin orthologs as well as two human protein variants (W100E and W100Q) which carry a substitution of the exposed hydrophobic Trp side chain at position 100 of the mature protein by a hydrophilic residue (Figure 1). The influence of these mutations on the potential immunogenicity of the modified protein in humans was assessed *in silico* using the IEDB deimmunization Web server (http://tools.iedb.org/main/tcell).^48^ Neither of the two side chain substitutions led to a new T cell epitope compared to the human wild-type leptin. All four recombinant proteins were produced in the cytoplasm of E. coli Origami B with an N-terminal His_6_-tag. Furthermore, the influence of a PAS-polypeptide^19^ comprising 200 amino acid residues, PAS(200), when fused with the N-terminus of each of the leptin versions was investigated.

**Figure 1:**
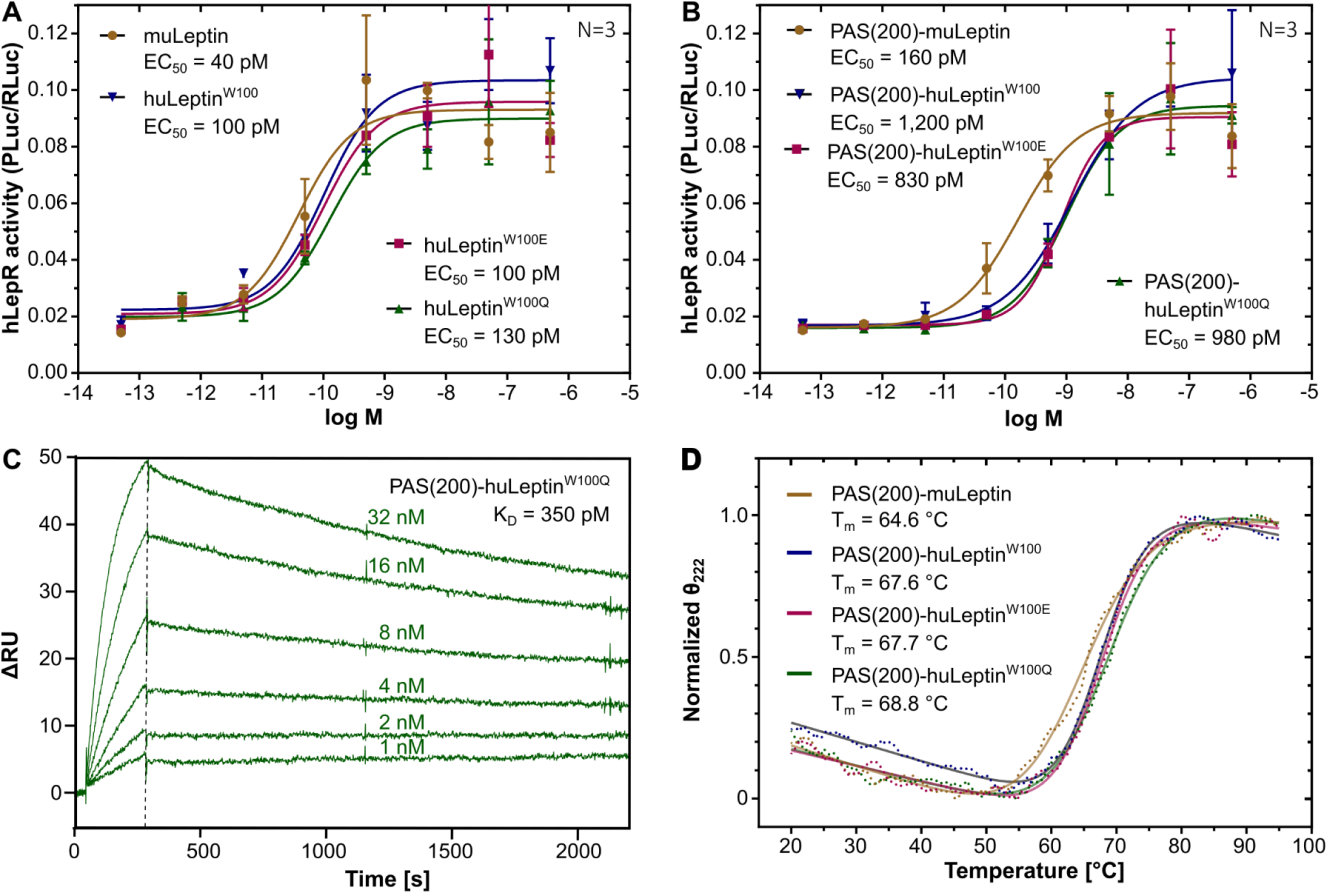
Biochemical analysis of human leptin variants constructed in this study. (A, B) Receptor activation on HEK cells transfected with the human LEPRb gene together with a STAT3-responsive Photinus luciferase and a constitutively expressed Renilla luciferase (for signal normalization). Cells were stimulated with (A) recombinant unmodified or (B) PASylated leptin variants (50 fM–500 nM), followed by measurement of JAK/STAT activation using a dual luciferase assay. (C) Real-time SPR binding analysis of PAS(200)-huLeptin^W100Q^ to its target human LepRb, which was immobilized as Fc-fusion protein on the sensorchip. (D) Thermal unfolding of PASylated leptin variants monitored by CD spectroscopy at 222 nm (curve fits shown as solid lines).

The biological potency was assessed in HEK293 cells overexpressing the long-form of the human leptin receptor gene, LEPRb, as well as a STAT3-responsive luciferase reporter gene.^49^ Typical sigmoidal dose–response-curves were observed for all leptin versions, resulting in half-maximal effective concentrations (EC_50_) of 40 pM for the murine leptin and of 100 pM, 100 pM, and 130 pM, respectively, for the human wild-type leptin and its mutants W100E and W100Q (Figure 1A). For the corresponding PASylated leptin variants elevated EC_50_ values of 160 pM, 1,200 pM, 830 pM, and 980 pM, respectively, were measured (Figure 1B). Taken together, the murine leptin showed slightly higher potency to activate the human LepRb compared to the human leptin and its mutants, which exhibited mutually similar activities. This relative pattern was unchanged for the PASylated leptin variants, although there was a moderate reduction in activity (which is, however, overcompensated by the much longer plasma half-life, see below) due to the attached voluminous PAS chain.^20^

These findings were complemented by real-time SPR interaction analyses of the leptin variants with a recombinant LepR-Fc fusion protein immobilized on the sensorchip (Figure 1C). In these measurements, the K_D_ values of PAS(200)-huLeptin^W100E^ and PAS(200)-huLeptin^W100Q^ were determined as 470 pM and 350 pM, respectively. Furthermore, the thermal folding stability of the PASylated leptin variants was analyzed by CD spectroscopy (Figure 1D). Here, all proteins exhibited a comparable melting temperature (*T*_m_), with 64.6 °C for murine leptin and 67.6–68.8 °C for the human leptin versions.

These data confirm that the murine and human leptin orthologs are comparable in their properties regarding receptor affinity and activation as well as protein folding stability. Based on these findings, the human leptin carrying the mutation W100Q was chosen as the lead compound, also considering the conservation of this amino acid in closely related species, such as rodents, pig, and cattle (see Figure S1), and data on solubility-enhancing point mutations of human leptin published by the pharmaceutical industry.^25,27^

### 3.2 Determination and Modeling of the Leptin PK in Mice

In order to better understand the concentration profile of a PASylated human leptin drug candidate in the blood during the time course of an experiment, we first determined the PK of the fusion protein, P/A(600)-huLeptin^W100Q^, utilizing a longer polypeptide with the more recently developed P/A#1 sequence, which was derived from the original PAS#1 sequence^19^ (sequence type ″#1″ from now on omitted for convenience) via replacement of all Ser residues by Ala.^32^ P/A(600)-huLeptin^W100Q^ was administered s.c. to wild-type C57BL/6J mice, resulting in a plasma half-life of 18.8 ± 3.6 h (Figure 2). This elimination half-life is essentially identical to the PK previously measured for the corresponding murine leptin fused with a PAS(600) polypeptide (19.6 ± 2.4 h) after intraperitoneal administration.^20^ Next, a one-compartment model with first-order absorption from the s.c. dosing site^38^ was adapted to simulate a repeated-dose study with *Lep* ^*ob/ob*^ animals using Berkeley Madonna software (Figures 2B and S4). Parameters for the clearance of the PASylated leptin (CL600) and the permeation from the dosing site to the blood compartment (*k*_a_) were obtained from the curve fit of the experimental PK data (Figure 2A), again using Berkeley Madonna, while the clearance value for the unmodified leptin was taken from the previous PK study of murine leptin^20^ and *k*_a_ was assumed to be the same.

**Figure 2:**
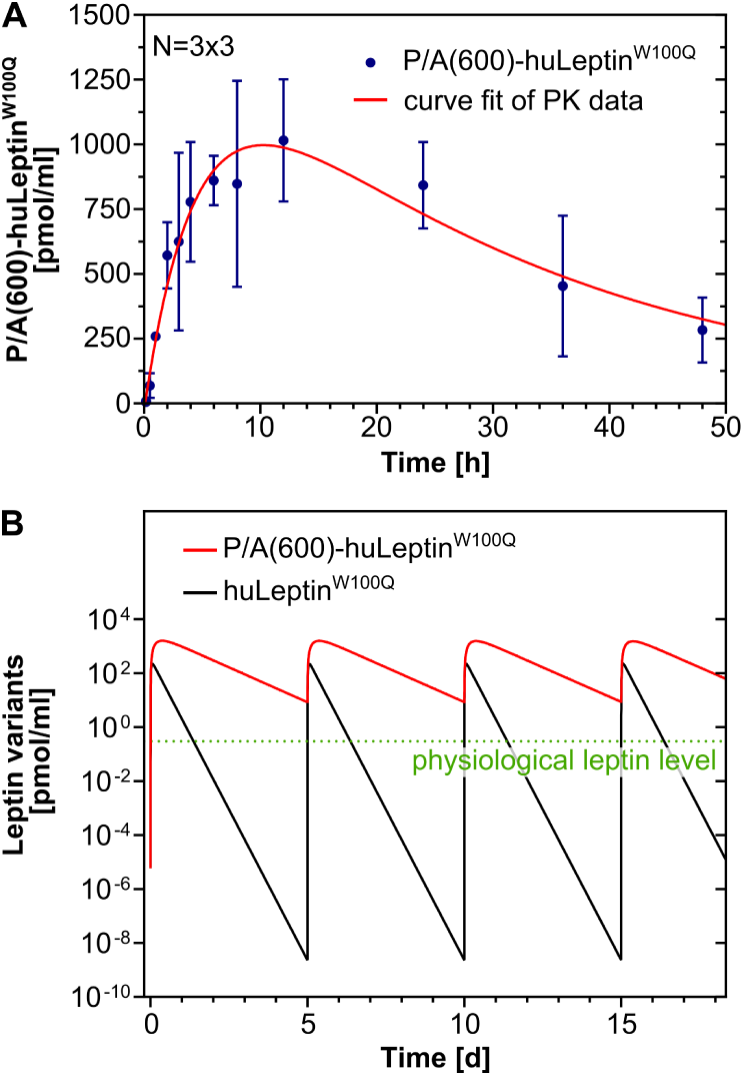
PK study of PASylated human leptin and one-compartment model with first-order absorption for repeated dosing. (A) PK profile of P/A(600)-huLeptin^W100Q^ after s.c. injection into ♂ wild-type C57/BL6 mice. Curve fit of the experimental PK data was performed with Berkeley Madonna using the variable parameters *k*_a_ (transport rate from the dosing site to the blood compartment), Cl (clearance from the blood compartment) and dose. (B) Repeated-dose PK simulation based on the parameters from (A).

For the PK simulation based on a system of ordinary differential equations, the anticipated b.w. changes of the heavily obese *Lep* ^*ob/ob*^ mice under treatment with huLeptin^W100Q^ or P/A(600)-huLeptin^W100Q^ were included, and the model was furthermore adapted to the altered blood volume of these animals, since the additional fat mass is less perfused. As expected, the maximal drug concentration in blood (*C*_max_) was much higher for P/A(600)-huLeptin^W100Q^ (∼1,600 pmol/mL) than for the non-PASylated huLeptin^W100Q^ (∼220 pmol/mL). Furthermore, the time point of the highest drug concentration (*t*_max_) was reached much earlier for huLeptin^W100Q^, after 1.5 h, than for P/A(600)-huLeptin^W100Q^, with *t*_max_ = 9.0 h. The difference between the non-PASylated leptin and the P/A(600)-huLeptin^W100Q^ was further illustrated in the repeated-dose simulation by the time period during which the plasma concentration stayed above the physiological leptin level of wild-type mice (0.3 pmol/mL; dotted green line in Figure 2B). Based on the experience from the previous PD study with *Lep* ^*ob/ob*^ mice^23^ and the predictions of this simulation, we set the injection interval to 5 days for a new animal treatment study, using a b.w.-adjusted dose, as this appeared to result in a continuous exposure to the PASylated leptin without building up increasing blood levels.

### 3.3 PD Study of *Lep* ^*ob/ob*^ Mice with PASylated Human Leptin

In a treatment study of *Lep* ^*ob/ob*^ mice, two different PASylation sequences were compared *in vivo*, PAS(600) and P/A(600), which were individually fused with huLeptin^W100Q^, this time also avoiding an affinity tag or other extraneous amino acid sequences (see Method section “Bacterial production and purification of leptin” and Figure S2). Both proline/alanine-rich sequences differ by the replacement of all Ser residues present in the PAS#1 sequence motif^19^ by Ala, resulting in the P/A#1 sequence which is composed of Ala and Pro residues only, whereby both the random coil conformation and the strong hydrophilicity of the polypeptide are retained.^32^ Furthermore, the results of the PK study above indicated that this minor change in the sequence does not measurably affect the plasma half-life of the leptin fusion protein. For comparison, two further cohorts were treated with the same molar dose of the non-PASylated recombinant human leptin or a vehicle control (PBS).

The repeated-dose study was performed with *Lep* ^*ob/ob*^ mice (*N* = 4) by s.c. administration of 100 pmol/g b.w. of the corresponding leptin version on days 0, 5, 10, and 15 (Figure 3). As expected, the animals showed drastically reduced food intake both with PAS(600)-huLeptin^W100Q^ and with P/A(600)-huLeptin^W100Q^ throughout the experiment as compared to the plain leptin and vehicle-treated mice (Figure 3A,B). Accordingly, the b.w. of animals treated with PAS(600)-huLeptin^W100Q^ or with P/A(600)-huLeptin^W100Q^ progressively declined by up to ∼50% toward the end of the study. In contrast, in both control groups the b.w. tended to increase slightly (Figure 3C), in line with the finding from previous *in vivo* experiments, which indicated that the low molar dose of the non-PASylated leptin applied here is insufficient to reduce food intake.^23^ Furthermore, both PASylated leptin versions led to an increased body temperature of 37.3 ± 0.5 °C (Figure 3D). For comparison, the body temperature of the control groups remained at 36.4 ± 0.7 °C (huLeptin^W100Q^) and 35.8 ± 0.7 °C (PBS), respectively, suggesting a UCP1-dependent increase in body temperature induced by leptin as previously described.^23^

**Figure 3:**
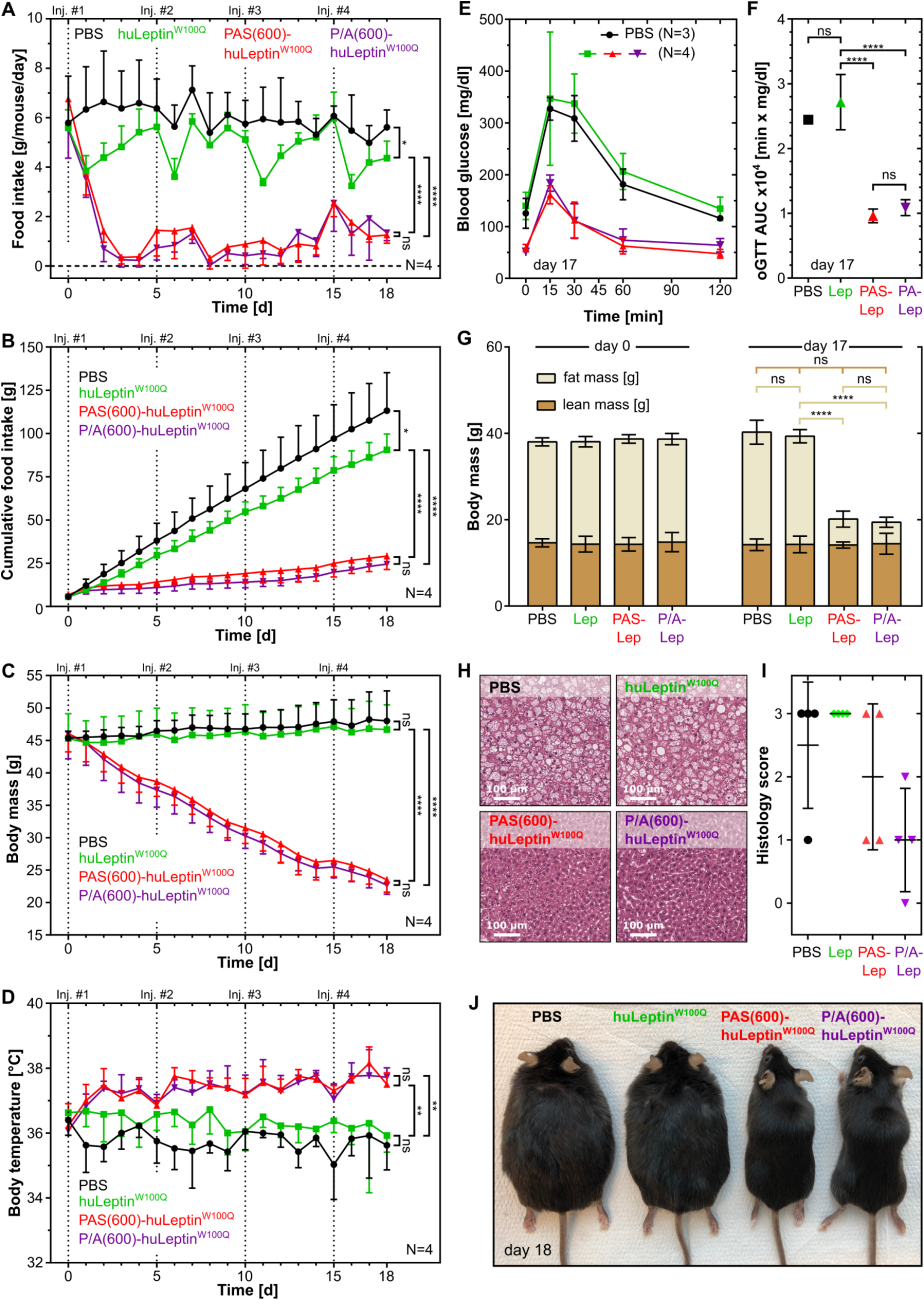
*In vivo* characterization of human leptin and its PASylated versions in mice. *In vivo* studies were performed with ♂ leptin-deficient *Lep* ^ob/ob^ mice that were treated with PAS(600)-huLeptin^W100Q^, P/A(600)-huLeptin^W100Q^ or huLeptin^W100Q^, with PBS injections as control. Cohorts of 4 mice each were treated with 100 pmol/g s.c. on days 0, 5, 10, and 15. (A) Daily and (B) cumulative food intake was analyzed for each animal, together with (C) b.w. and (D) body temperature. One day before the end of the study (day 17), oral glucose tolerance was assessed (E, F, color code as in A), and the body composition was investigated using NMR (G). At the end of the study (day 18), hepatic steatosis was analyzed by HE-staining (H) and scored by a blinded expert pathologist (I). (J) Treatment outcome is illustrated by a photograph of representative animals from each cohort at the end of the study.

Oral glucose tolerance was tested toward the end of the treatment (day 17) and indicated a normalized response (Figure 3E), which was reflected by a 2.2- to 2.5-fold reduction in the oGTT AUC for mice treated with one of the PASylated leptin versions (Figure 3F) compared with the other two groups. NMR analysis of the body composition revealed that the drastic overall loss in b.w. upon treatment with both PASylated leptin versions was predominantly due to a reduction in fat mass. In contrast, the lean mass remained constant (Figure 3G). This selective fat loss was also seen at the cellular level by analyzing HE-stained hepatic sections (Figures 3H and S6). Hepatic steatosis was scored on a scale from 0 (no steatosis) to 3 (severe steatosis) and revealed a considerable improvement for the *Lep* ^*ob/ob*^ mice treated with PAS(600)-huLeptin^W100Q^ or P/A(600)-huLeptin^W100Q^ (Figure 3I).

At the end of the 18 day study with both PASylated leptin versions, the morbidly obese mice had assumed the normal appearance of a lean mouse (Figure 3J). Thus, the treatment with only four injections of a PASylated leptin—independent of the precise amino acid sequence of the PAS polypeptide—resulted in a complete reversal of the *Lep* ^*ob/ob*^ phenotype, which could not be achieved by injection of the unmodified leptin at the same molar dose (and a 5-day dosing interval). None of the measured parameters showed a significant difference between the PAS(600)-huLeptin^W100Q^ and P/A(600)-huLeptin^W100Q^ treated groups, thus indicating similar functionality of both PASylation sequences. Accordingly, this supports the purely biophysical mechanism of an expanded hydrodynamic molecular volume^32^ as explanation for the extended plasma half-life and the drastically enhanced PD effect of the PASylated leptin seen here. Of note, the P/A sequence, which consists of Pro and Ala residues only, lacks any potentially reactive amino acid side chains (including the weakly nucleophilic Ser) and in this regard resembles even more closely the chemically inert polyethylene glycol (PEG), a synthetic polymer that is widely used to prolong the PK of biological drugs.^50^ Hence, the P/A polypeptide was chosen to further investigate the metabolization of the PASylated human leptin mutant in mice.

### 3.4 Design of a Doubly Labeled Fluorescent PASylated Leptin Imaging Probe

PASylated murine leptin was previously shown to stimulate STAT3 phosphorylation in the hypothalamus of mice,^20^ which is protected from normal circulating proteins by the BBB and other barriers within the CNS, in line with an active import mechanism for leptin.^8,11^ However, the question remained open if the PASylated leptin passes these barriers in an intact state or if the natively unfolded PAS-polypeptide may be cleaved during transcytosis, e.g., by endosomal proteases.^51^ To study the stability and fate of the PASylated leptin *in vivo*, we constructed a molecular probe that comprises two different regio-specifically attached near-infrared fluorescent labels, one at the N-terminus of the P/A(200) sequence and another one at the leptin moiety (Figure 4).

**Figure 4:**
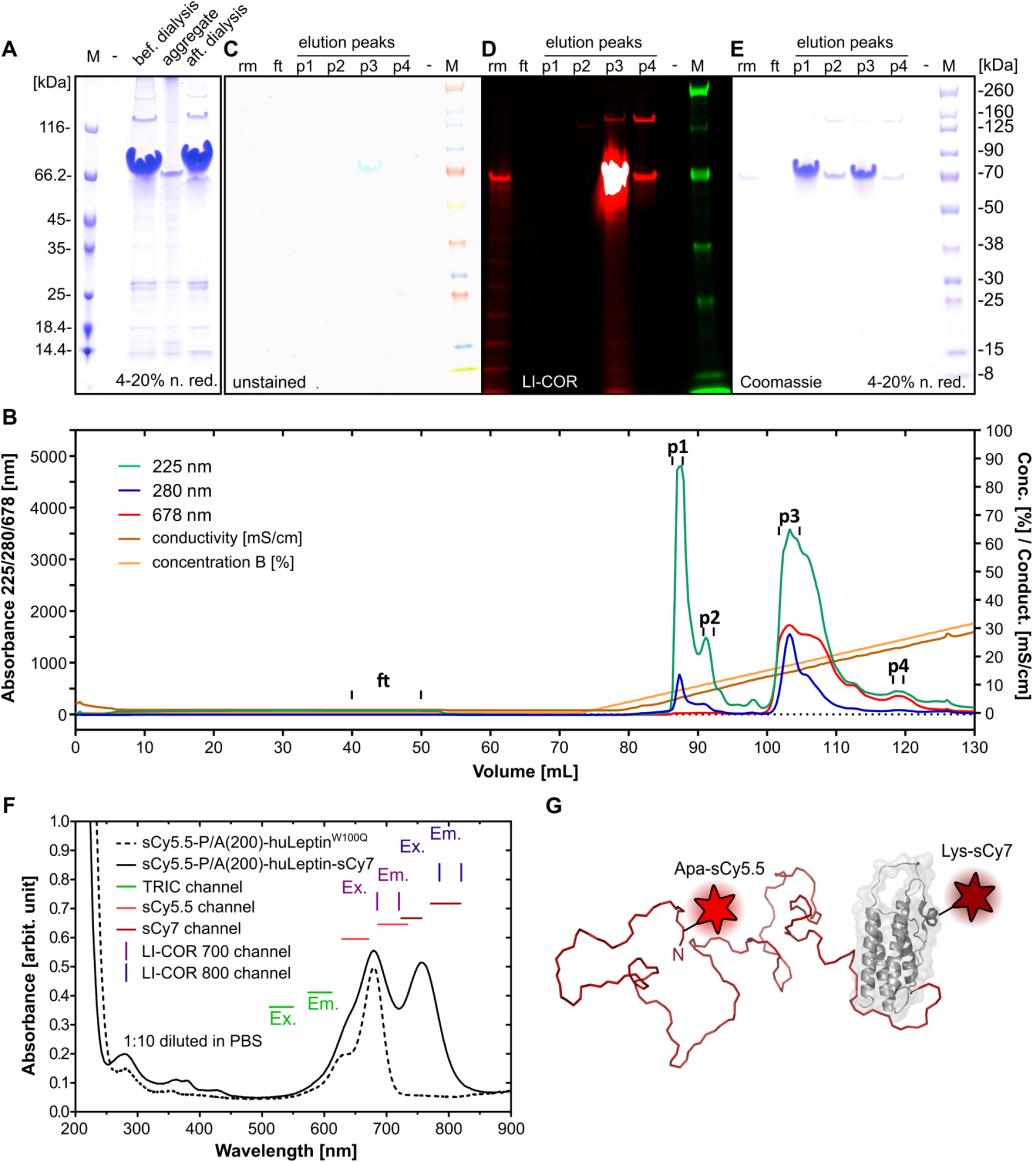
Preparation of the doubly fluorescent sCy5.5-P/A(200)-huLeptin^W100Q^-sCy7 imaging probe. The fusion protein MP-His_6_-Apa-P/A­(200)-huLeptin^W100Q^ was produced in E. coli Origami B using a plasmid system for the site-directed incorporation of the non-canonical amino acid Apa close to the N-terminus via amber stop codon suppression. The protein was purified by IMAC, dialyzed against a slightly acidic buffer (A) and, subsequently, conjugated with the keto-reactive dye, hydrazide-sCy5.5. In a subsequent AEX purification step, the additional negative charges of the dye permitted the isolation of the conjugated protein (B). The AEX peaks (p1–p4) were analyzed by SDS-PAGE (rm, reaction mixture prior to AEX; ft, flow through; M: Chameleon Duo pre-stained protein size marker) using a transmission light scan (C), a fluorescence scan with a LI-COR Odyssey instrument (700 nm channel red, 800 nm channel green) (D), as well as Coomassie staining (E). The AEX allowed the baseline-separation of the unconjugated protein (p1-p2) from the sCy5.5-conjugated PAS-huLeptin product (p3). The sCy5.5-conjugated PASylated leptin was subsequently conjugated at a ∼1:1 ratio with the amino-reactive dye NHS-sCy7, which was mediated by one of the in total seven Lys residues that exclusively occur in the leptin moiety, followed by final SEC. (F) UV/Vis spectroscopic analysis after the first and second dye-coupling reactions indicated a 1:1:0.9 conjugation ratio in the sCy5.5-P/A(200)-huLeptin^W100Q^-sCy7. (G) 3D model of the doubly fluorescent sCy5.5-P/A(200)-huLeptin^W100Q^-sCy7 imaging probe.

The site-specific conjugation of P/A(200)-huLeptin^W100Q^ with fluorescent dyes at either side of the PAS polypeptide, resulting in sCy5.5-P/A(200)-huLeptin^W100Q^-sCy7 (Figure 4G), required a bio-orthogonal conjugation strategy that allowed a precise 1:1:1 coupling ratio while preserving solubility of the aggregation-sensitive leptin moiety. To this end, we produced a biosynthetically modified protein, Met-Pro-His_6_-Apa-P/A­(200)-huLeptin^W100Q^, using an E. coli amber suppression system (Figure S3).^45^ In this way, the noncanonical amino acid *p*-acetyl-l-phenylalanine (Apa), which offers a keto function in its side chain for bio-orthogonal conjugation, was incorporated close to the N-terminus of the fusion protein (see Figure S3).

Starting from the purified biosynthetic protein, the chemical coupling of the Apa side chain with two different dyes was compared: aminooxy-sCy5 and hydrazide-sCy5.5, which readily form oxime and hydrazone linkages, respectively, with the keto group of the Apa side chain in aqueous milieu (Figure S7). Different pH values were investigated to find the ideal reaction conditions, indicating a strong pH-dependence, with optimum at pH 4.5 for both dye derivatives (see Figure S7C–E). Purification of the conjugates was achieved by AEX on a Resource Q column, which, however, resulted only in partial separation of the sCy5-aminooxy conjugate from the unconjugated protein (Figure S8). In contrast, in case of the sCy5.5-hydrazide, which carries four (instead of two) sulfonate substituents, baseline separation of the dye conjugate from the unlabeled protein was achieved due to its enhanced electrostatic interaction with the chromatography matrix.

Finally, the chemical stabilities of the hydrazone- and oxime-linkages were tested *in vitro* by challenging both conjugated leptin fusion proteins for 6 h at 37 °C via incubation with 1:1 (v/v) murine serum (Figure S9) or with 500 mM Na-acetate pH 5.5—in an attempt to mimic the lower pH in endosomal vesicles, such as during transcytosis. Both linkages showed comparable stability, with >95% of the dye-protein conjugate remaining intact. Due to the more efficient isolation, we chose the hydrazide-sCy5.5 dye for preparative conjugation with P/A(200)-huLeptin^W100Q^ (Figure 4).

After preparative coupling and purification by AEX (Figure 4B), the conjugate was analyzed by SDS-PAGE and transmitted light scanning (Figure 4C), fluorescence scanning (Figure 4D) as well as Coomassie staining (Figure 4E). The resulting homogeneous sCy5.5-P/A(200)-huLeptin^W100Q^ conjugate was then reacted with sCy7-NHS to achieve coupling via the ε-amino group of one of the seven Lys residues within the leptin moiety. Due to the statistical nature of this coupling step the receptor-binding activity of the majority of the leptin domain should be maintained while yielding dye labeling approximately at a 1:1 ratio. Of note, the Pro residue chosen for the N-terminus of the P/A moiety served to avoid conjugation at this site (as a side reaction) due to the much reduced nucleophilicity of its imino nitrogen. Final absorption spectroscopy confirmed a conjugation ratio of 0.9 sCy7 groups per sCy5.5-P/A(200)-huLeptin^W100Q^ molecule (Figure 4F), thus yielding the doubly labeled sCy5.5-P/A(200)-huLeptin^W100Q^-sCy7 probe (Figure 4G).

### 3.5 *In Vivo* Imaging of the Fluorescent PASylated Leptin via Light Sheet Fluorescence Microscopy (LSFM)

The doubly labeled imaging probe from above was injected i.v. into C57BL/6J mice via the tail vein at a dose of ∼250 μg per animal. Distribution, metabolization and elimination of sCy5.5-P/A(200)-huLeptin^W100Q^-sCy7 were allowed for 8 h. Due to the increased plasma half-life of the PASylated leptin, it was expected that a sufficient portion of the injected protein would have remained in the blood circulation at this point. Fifteen min prior to the end of the experiment, a lectin conjugated with a spectrally distinguishable Dy-594 dye was injected in order to stain the blood vessels. After perfusion with ice-cold PBS and PAXgene tissue fixative, the kidneys, liver, brown adipose tissue (BAT), and the brain were dissected and optically cleared using the 3DISCO method.^47^ After that, the three fluorescent dyes were individually detected via LSFM using corresponding wavelength channels (Figures 5 and 6).

**Figure 5:**
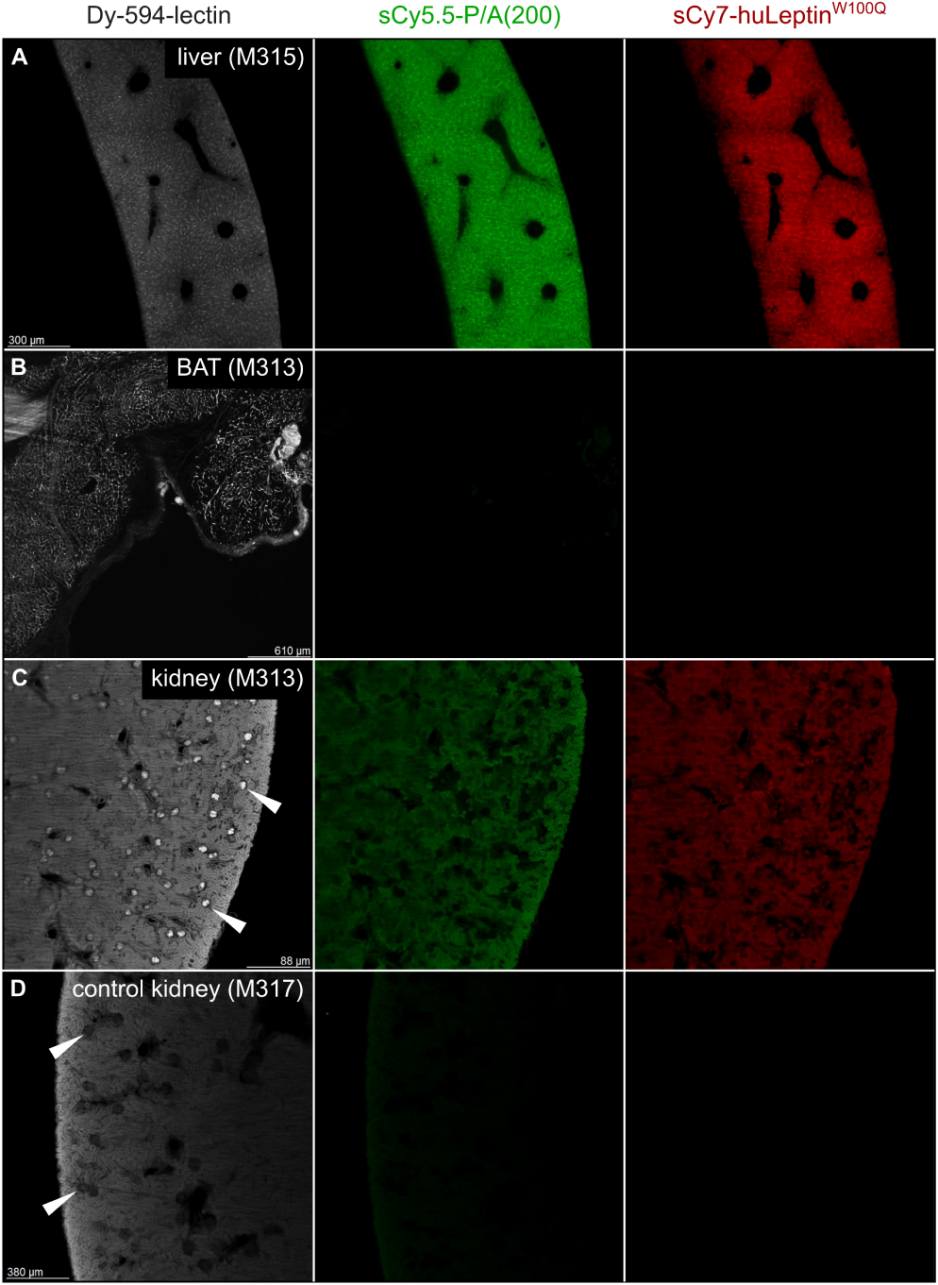
Light sheet fluorescence microscopy after *in vivo* application of the doubly fluorescent PASylated leptin imaging probe. C57BL/6J mice (♀) were injected i.v. with the sCy5.5-P/A(200)-huLeptin^W100Q^-sCy7 imaging probe at 300 pmol/g b.w. and sacrificed at *t* = 8 h post injection by perfusion with PAXgene. Fifteen min prior to that, 3 nmol Dy-594-lectin was injected i.p. in order to stain the blood vessels and capillaries. Organs were dissected, fixed with PAXgene, and cleared using the 3DISCO method. For each organ, one representative z-plane is depicted in three panels corresponding to the fluorescence signals from the Dy-594-lectin (left column, white), the sCy5.5-P/A(200) moiety (middle column, green), and the huLeptin^W100Q^-sCy7 moiety (right column, red). Organs shown include (A) liver, (B) brown adipose tissue (BAT), (C) a kidney and (D) a kidney from a not-injected mouse (only revealing some autofluorescence of the tissue in different channels). White arrow heads indicate glomeruli within the kidney.

**Figure 6:**
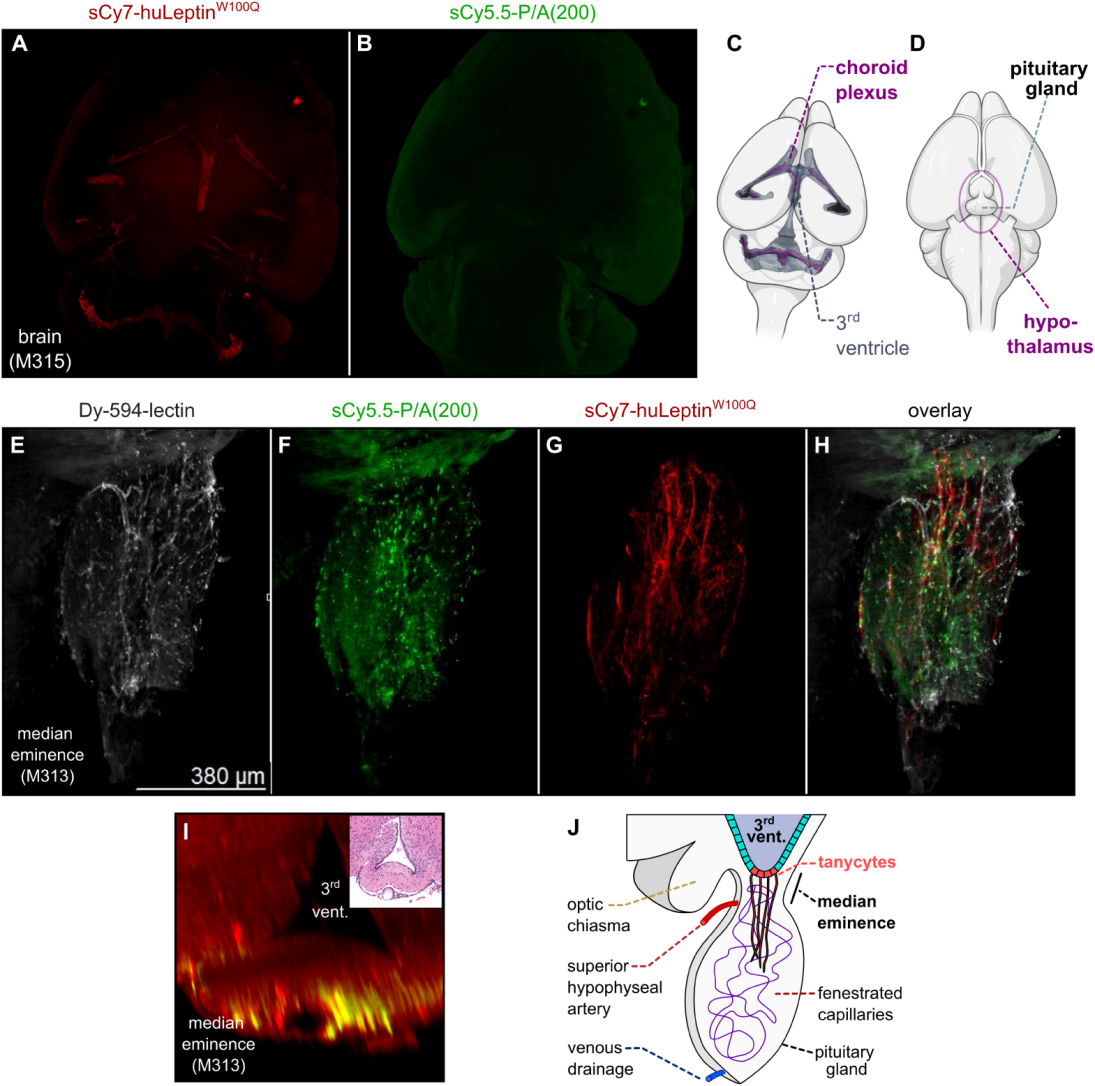
Cleavage of the PASylated leptin imaging probe within the median eminence (ME) of the hypothalamic brain region. (A, B, E–H) Maximum intensity projections (MIP) of mouse brains treated with the sCy5.5-P/A(200)-huLeptin^W100Q^-sCy7 imaging probe as in Figure 5. (A, B) Dorsal MIP of the mouse brain. (C) Schematic dorsal view depicting the CP within the mouse brain (taken in part from Greiner et al., 2022).^52^ (D) Schematic ventral view of a mouse brain showing the hypothalamus and the pituitary gland. (E–H) Ventral MIP of the stalk of the pituitary gland (partly fractured during the preparation) close to the ME, imaged in individual fluorescence channels for the Dy-594-lectin (E), sCy5.5-P/A(200) (F), huLeptin^W100Q^-sCy7 (G), and depicted as an overlay (H). (I) Coronal image plane derived from a 3D model showing the distribution of fluorescence signals within the ME region. Inlet: Coronal H&E stained paraffin section of the sample shown. (J) Lateral schematic section through the ME and the pituitary gland. Panels (C) and (D) were in part created using BioRender.com.

In the kidneys, the glomeruli were intensely stained by the fluorescent lectin. While the kidney of the mouse injected with the bifluorescent PASylated leptin probe showed a nearly uniform accumulation, which was indicated by the detection of both fluorophores (Figure 5C), corresponding signals were absent in the kidney of an untreated control mouse (Figure 5D). In the liver, the two fluorescence signals from the PASylated leptin appeared colocalized and associated with cellular structures, potentially Kupffer cells (Figure 5A). In contrast, the branched vasculature in BAT was stained with the lectin, but no signal indicative of the labeled leptin probe was seen (Figure 5B).

While at least some of the peripheral organs showed colocalized fluorescence signals, as expected for the doubly labeled intact PASylated leptin, tissue samples from the CNS indicated a cleavage of the fusion protein since signals from sCy5.5 and sCy7 were not colocalized (Figure 6). Interestingly, the dorsal maximum intensity projection (MIP) of the brain revealed a local accumulation of the sCy7-labeled huLeptin^W100Q^ moiety in the ventricle, distributed in a pattern resembling the CP (Figure 6B), but, surprisingly, no signal for the sCy5.5-labeled P/A(200) polypeptide (Figure 6A). In fact, the stained anatomical structure closely matches published images^52^ of the murine CP (Figure 6C). Leptin signals in the CP are likely associated with its transport across the BCSFB there.^8^

Another interesting observation appeared from a more focused MIP view in the region of the ME and the stalk plus posterior lobe of the (seemingly truncated) pituitary gland (Figure 6D).^53^ In this hypothalamic brain region, signals were detected in all three fluorescence channels (Figure 6E–H). However, the fluorescence signals specific for the leptin moiety from the PASylated leptin probe were mostly not colocalized with those of the PASylation tag. While the sCy5.5 signal corresponding to the P/A(200) polypeptide appeared localized in spots (Figure 6F), the sCy7 signal of the huLeptin^W100Q^ moiety emerged in unidirectional linear structures (Figure 6G). Notably, the correlation of fluorescence signals at different z-levels showed that this leptin signal mostly did not colocalize with that of the Dy-594-lectin-stained larger vessels (Figure 6H). These leptin-sCy7 signals in the stalk of the pituitary gland appeared near the ME and the third ventricle, as can be seen from coronal image planes derived from a 3D reconstruction (Figure 6I). This indicates a potential processing of the sCy5.5-P/A(200)-huLeptin^W100Q^-sCy7 fusion protein during the leptin transport process across the tanycytic barrier (Figure 6J). Tanycytes are highly specialized ependymal cells that form their own barrier at the level of the ME and were previously shown to transport leptin into the mediobasal hypothalamus.^54,55^ Tanycytes also express LepRb and are able to sense leptin.^11,46,56^

## 4 Discussion

### 4.1 Biochemical Comparison of Human Leptin Mutants

The previously described murine leptin version with prolonged plasma half-life, PAS(600)-muLeptin,^20,22,23^ had demonstrated efficacy in different murine disease models and shown potential to unlock a novel route toward the development of a promising drug candidate.^57–59^ To optimize a therapeutically viable human leptin version with reduced aggregation propensity—due to the naturally exposed Trp side chain at position 100—we compared the hydrophilic amino acid substitutions W100E and W100Q with regard to folding stability and activation of LepRb. Both leptin variants activated the human leptin receptor with a comparable EC_50_ value of 100–130 pM, which matches the published value of 150 pM for native leptin.^49^ Due to its prevalence in other mammalian species, the Gln residue at position 100 was chosen for further development.

### 4.2 Evaluation of PD Efficacy for the PASylated Human Leptin

To investigate *in vivo* efficacy in a repeated dose study in *Lep* ^*ob/ob*^ mice, the comparison of two previously established PASylation sequences, PAS#1 and P/A#1,^32^ was of interest. Both PASylation sequences comprise repeated 20-residue stretches with a Pro content of 35%. While PAS#1, (ASPAAPAPASPAAPAPSAPA)_n_, contains three Ser residues per repeat motif, all these positions were replaced by Ala in the P/A#1 sequence, (AAPAAPAPAAPAAPAPAAPA)_n_. This results in a chemically extremely robust biopolymer without any side chain reactivities, thus fully preventing modifications both *in vivo* and *in vitro* (e.g., during drug storage). Although both proline/alanine-rich sequences (PAS) have been shown to adopt robust random coil conformation under physiological buffer conditions and exhibit very similar biophysical properties, including strong hydrophilicity,^32^ it was of interest to determine if there is any difference in the PD efficacy of corresponding leptin fusion proteins.

Interestingly, the results of this study revealed indistinguishable *in vivo* activity between both huLeptin^W100Q^ fusion proteins, which confirms the similar polymer behavior of the PAS and P/A sequences as demonstrated *in vitro* before.^32^ In particular, our data prove high bioavailability of the P/A-leptin fusion protein even after s.c. injection, which provides an advantage over PEGylated drugs that often show delayed dose absorption when combined with the subcutaneous route of administration.^60^ Furthermore, the PD effects seen here for both PASylated human leptin versions are in line with a previous study on the murine PASylated leptin, employing the PAS#1 sequence, where four doses of the PAS-leptin fusion protein also led to a complete reversal of the *Lep* ^*ob/ob*^ phenotype.^23^

As an alternative to leptin replacement therapy, the monoclonal human IgG antibody mibavademab (REGN4461), which activates human LepRb in the presence or absence of leptin, was developed.^61^ Recently, this drug candidate proved effective in a lipodystrophy patient with a recorded history of anti-leptin antibodies as part of a compassionate use treatment.^61,62^ Notably, mibavademab features the distribution profile of an intact antibody, thus presumably lacking the capacity for efficient CNS delivery. Its preclinical evaluation included treating leptin-deficient mice expressing a humanized extracellular LepR domain (*LepR* ^*hu/hu*^ *Lep* ^–/–^), similar to the *Lep* ^ob/ob^ animal model used in the present study. While mibavademab treatment led to 33.7% b.w. reduction within 38 days,^62^ treatment with P/A(600)-huLeptin^W100Q^ was more than twice as effective in the present study, leading to >50% b.w. reduction within only 18 days. The higher biological activity of PASylated huLeptin^W100Q^ may be explained by more efficient leptin signaling triggered within the CNS.

The drastic plasma half-life extension of leptin achieved by PASylation should prolong its dosing interval at least to weekly injections in patients (considering the rules of allometric scaling from mice to man). Increased solubility during liquid formulation or reconstitution of the lyophilized drug, as well as considerably reduced aggregation tendency as a result of the W100Q substitution, together with the hydrophilic shielding properties of the attached PAS polypeptide, should contribute to a much reduced immunogenicity—as already proven after repeated dosing in a murine disease models^23^ which is a known caveat for the approved drug metreleptin.^29,63,64^

### 4.3 Cleavage of the PASylation Tag During CNS Targeting

While intracellular proteases quickly digest PAS sequences—thus providing an advantage over the nonbiodegradable PEG—these polypeptides have proven full stability in blood.^19,65^ As leptin exerts central nervous effector functions within the hypothalamus, it may serve as a test system for the potential metabolization of PASylated proteins during transcytosis from the blood. Although the mechanism by which leptin enters the CNS is not fully understood so far, a blood-brain shuttle system involving tanycytes was recently described.^55,66^ Tanycytes are specialized glial cells located in the ME region of the hypothalamus,^67^ where they line the third ventricle wall with processes reaching into the ME. Leptin appears to leave the blood via fenestrated capillaries within the ME, where it gets bound by LepR located on these processes, followed by transcytotic transport into the third ventricle.^54,55^

The proven CNS efficacy of PASylated leptin *in vivo* does not provide information on the integrity or potential metabolization of the fusion protein outside the blood compartment (note that cleavage within the plasma would lead to a shortened half-life as well as truncated protein fragments that would be detectable on a Western blot).^65^ In order to study the fate of PASylated leptin in the brain as well as other physiologically relevant organs or tissues, a PASylated leptin probe with spectrally distinguishable fluorescent dyes attached to both moieties of the fusion protein was constructed.

As the complete formation of the single structural disulfide bridge within leptin is crucial, introducing an additional free Cys residue—as often used in conjunction with maleimide coupling chemistry—was avoided. Instead, the non-canonical amino acid *p*-acetyl-l-phenylalanine (Apa) was incorporated via amber suppression in E. coli.^45^ Its unique and chemically bio-orthogonal keto group was subsequently used to specifically couple a fluorescent dye to this position at the amino-terminal end of the PAS chain. After that, the leptin moiety was additionally conjugated with NHS-sCy7. The preparation of the highly pure doubly labeled sCy5.5-P/A(200)-huLeptin^W100Q^-sCy7 was achieved in an efficient manner at the milligram scale.

### 4.4 Tracking Leptin Transport and Cleavage of the PASylation Sequence from the Fusion Protein

The distribution, metabolization, and excretion of unmodified recombinant leptin in mice were previously studied using positron emission tomography (PET)^68^ and LSFM.^46^ While labeling of leptin with a positron-emitting radioisotope offers the possibility of noninvasively determining the macroscopic accumulation in a quantitative and time-dependent manner, labeling with a fluorescent dye allows investigation of the distribution pattern down to the cellular or even subcellular resolution *post mortem.* ^46^ In our LSFM study with the sCy5.5-P/A(200)-huLeptin^W100Q^-sCy7 probe we found specific signals within the cortex of the kidney, which indicates some accumulation in this organ. It is known that leptin is filtered in the renal cortex and reabsorbed by the tubular cells, followed by metabolization;^68^ in fact, the kidneys are responsible for >80% of physiological leptin elimination from the blood.^69^

Likewise, some uptake of the PASylated leptin was seen in the liver. There, the intact fusion protein potentially accumulated in Kupffer cells, in the region of the liver sinusoidal endothelial cells (LSECs) which govern the filtration processes in this organ. Apart from that, the liver also belongs to the organs that are known to express LepR.^7,70^ Interestingly, although LepR-dependent uptake in fat tissue was previously reported,^68^ we could not detect signals for the PASylated leptin in the BAT that was analyzed. Thus, the elevated body temperature induced by the PASylated leptin variants may involve central activation of sympathetic nerves and not result from direct activation of LepR within the BAT.^68,71,72^

In line with the notion that the brain is the central place for leptin signaling,^14,57^ its LepR-dependent accumulation was demonstrated in previous PET experiments,^68^ and three transport mechanisms for leptin, vascular BBB, BCSFB, and tanycytic barriers, were described as mentioned above.^8,46,56^ Interestingly, the two distinct fluorescence signals of the doubly labeled PASylated leptin were not colocalized in the brain (contrasting to the signals seen in the liver). This indicates a fragmentation of the fusion protein within the CNS. In fact, the signal in the CP was dominated by the leptin-sCy7 moiety of the fusion protein, whereas a signal for sCy5.5-P/A(200) was not detectable. This suggests cleavage and/or degradation of the PASylation sequence either during the transport across the BCSFB into the CP and/or during passage across the tanycytic barrier. Of note, the CP located within the ventricular system is the primary target of leptin, as it was demonstrated by intracerebroventricular (i.c.v.) injections of leptin which immediately activated all leptin-responsive hypothalamic neurons.^73^ However, the identity of dedicated receptors that mediate leptin transport across these barriers (including leptin receptor isoforms and low density lipoprotein receptor-related protein 2 *alias* megalin or epidermal growth factor receptor as candidates) is still a matter of debate.^6,55^

Indeed, discrete signals were seen in the ME, a brain area close to the hypothalamus where the tanycytic barrier is located. The vasculature of the ME is leaky (not BBB-like) and allows leptin to enter the extracellular space, from where tanycytic transport into the CSF and the arcuate nucleus is possible.^8^ Notably, in contrast to the CP, the ME showed fluorescence signals from both fluorophores, yet with distinct patterns. While the leptin-sCy7 signal was in part associated with smaller (but not larger) capillaries, as indicated by colocalization with the Dy-594-lectin stain, the sCy5.5-P/A(200) signal was more focal, which could indicate intracellular accumulation during the LepR-mediated transcytosis across the tanycytic barrier into the CSF. While the observed distribution of the PASylated leptin is in line with previous reports on non-PASylated leptin,^46,55^ it remains elusive how exactly this fusion protein enters the ventricular system, presumably involving the tanycyte shuttle process.^46,54,55^

Together with the strong PD effect seen for the PASylated human leptin in mice, our data suggest that PASylation offers an ideal technology for delivering pharmacological cargoes with extended plasma half-life into the CNS, across the vascular BBB, the BCSFB and/or the tanycytic barrier. PASylation would keep the drug in circulation, leading to a prolonged contact time with receptors to traverse these barriers, while, on the other hand, the drug may get released in an essentially unmodified state into the brain tissue and target its neuronal receptor there.

## Supplementary Material



## Notes

Sequence alignment of leptin orthologs among prominent mammals (Figure S1); coding region for the fusion protein MP-P/A(600)-huLeptin^W100Q^ cloned on pASK37 (Figure S2); coding region for the fusion protein His_6_-Apa-P/A­(200)-huLeptin^W100Q^ cloned on pSB8 (Figure S3); code used in Berkeley Madonna software for PK simulation and for curve fitting of the experimental PK data (Figure S4); purification of PASylated leptin from an E. coli whole cell extract using (NH_4_)_2_SO_4_ precipitation (Figure S5); histological analysis of liver tissue from leptin-treated *Lep* ^*ob/ob*^ mice by hematoxylin and eosin staining and periodate-Schiff reaction (Figure S6); conjugation of ketone-reactive dyes to the non-canonical Apa side chain in His_6_-Apa-P/A­(200)-huLeptin^W100Q^ (Figure S7); isolation of the keto-conjugated PASylated leptin by AEX, taking advantage of the sulfonate groups (Figure S8); stability assessment of the keto-conjugated fluorescent PASylated leptin (Figure S9) (PDF)
