# Supplementary material for: Long-Acting Human PASylated Leptin Reaches the Murine Central Nervous System and Offers Potential for Optimized Replacement Therapy
Source: Mol Pharm. 2025 May 7;22(6):3017–32. doi: 10.1021/acs.molpharmaceut.4c01503 (PMC12135040; doi:10.1021/acs.molpharmaceut.4c01503)
Supplement: Supplementary file 1 [file mp4c01503_si_001.pdf]

Supplementary Information

Long-acting human PASylated leptin reaches the murine central nervous system and offers potential for optimized replacement therapy

Volker Morath, Stefanie Maurer, Annette Feuchtinger, Rebecca Walser, Martin Schlapschy, Florian Bolze, Thomas Metzler, Johanna Bruder, Katja Steiger, Axel Walch, Martin Klingenspor, Arne Skerra

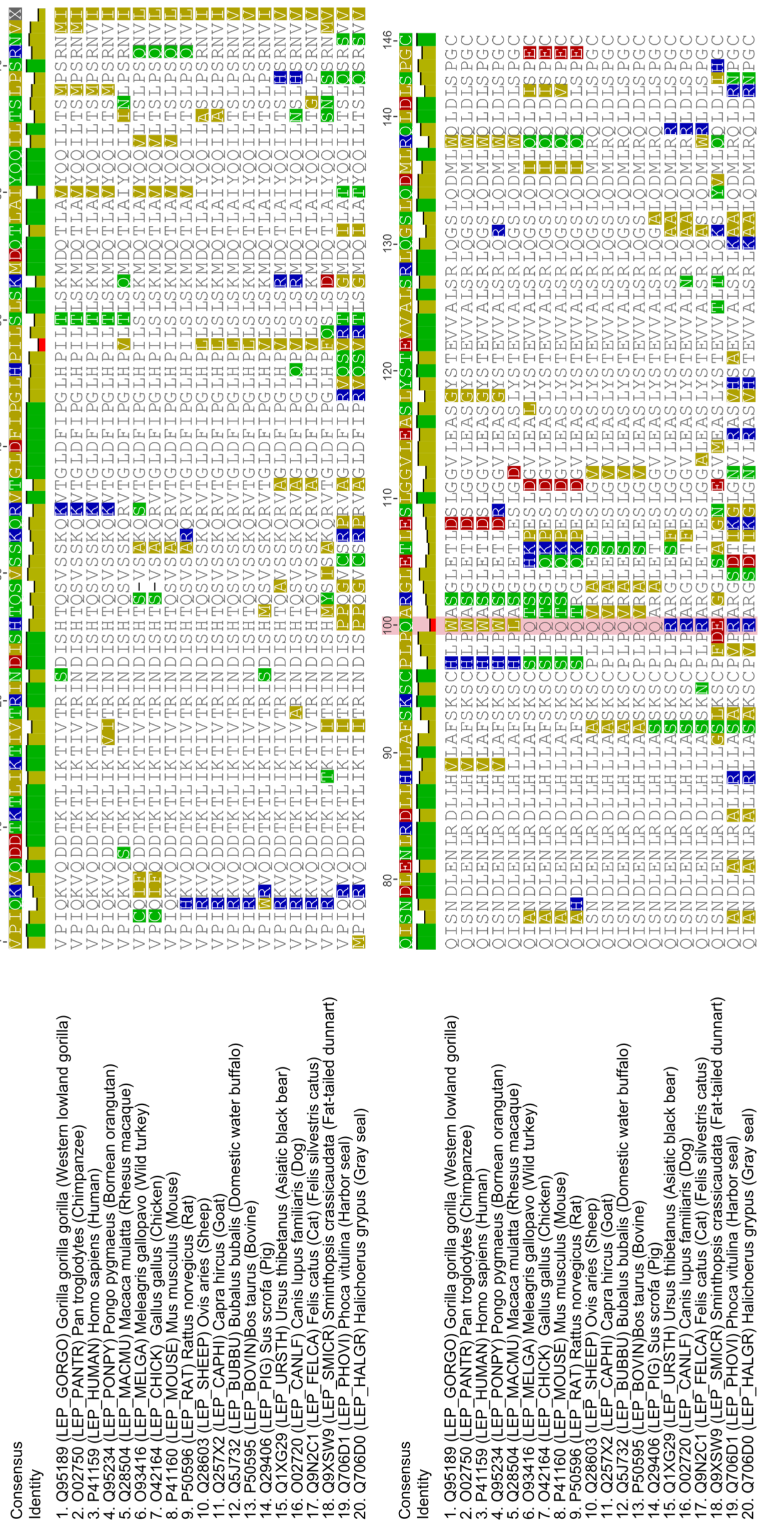

Figure S1: Sequence alignment of leptin orthologues among prominent mammals. Sequences were extracted from the UniProtKB database (https://www.uniprot.org) and aligned with ClustalW software using a BLOSUM cost matrix (opening: 20; extension: 1). Position 100 in the mature protein sequence is highlighted by a translucent red bar.

Human PASylated leptin

```
      XbaI                                  NdeI
   1 TCTAGAAATAATTTTGTTTAACTTTAAGAAGGAGATATACATATGCCAGCCGCTCCTGCT 60
                                               MetProAlaAlaProAla

  61 GCCCCTGCTCCCGCTGCCCCCGCCGCCCCCGCCCCAGCTGCCCCCGCTGCCGCACCTGCT 120
     AlaProAlaProAlaAlaProAlaAlaProAlaProAlaAlaProAlaAlaAlaProAla

 121 GCCCCAGCTCCCGCTGCCCCAGCCGCGCCGGCCCCCGCAGCTCCAGCCGCGGCACCAGCT 180
     AlaProAlaProAlaAlaProAlaAlaProAlaProAlaAlaProAlaAlaAlaProAla

 181 GCCCCAGCTCCAGCGGCGCCTGCTGCCCCGGCCCCCGCGGCACCGGCTGCCGCGCCCGCA 240
     AlaProAlaProAlaAlaProAlaAlaProAlaProAlaAlaProAlaAlaAlaProAla

 241 GCTCCAGCGCCTGCTGCACCGGCTGCTCCGGCACCCGCCGCGCCAGCAGCTGCCCCTGCG 300
     AlaProAlaProAlaAlaProAlaAlaProAlaProAlaAlaProAlaAlaAlaProAla

 301 GCACCAGCTCCTGCTGCCCCCGCGGCACCTGCACCCGCTGCCCCGGCGGCAGCTCCCGCC 360
     AlaProAlaProAlaAlaProAlaAlaProAlaProAlaAlaProAlaAlaAlaProAla

 361 GCGCCAGCCCCTGCAGCTCCTGCTGCACCTGCTCCTGCCGCCCCTGCTGCTGCCCCTGCT 420
     AlaProAlaProAlaAlaProAlaAlaProAlaProAlaAlaProAlaAlaAlaProAla

 421 GCTCCAGCCCCTGCAGCACCGGCCGCTCCAGCTCCTGCCGCTCCTGCCGCTGCGCCCGCT 480
     AlaProAlaProAlaAlaProAlaAlaProAlaProAlaAlaProAlaAlaAlaProAla

 481 GCTCCAGCCCCAGCTGCGCCAGCAGCTCCTGCACCTGCTGCCCCTGCCGCCGCCCCTGCG 540
     AlaProAlaProAlaAlaProAlaAlaProAlaProAlaAlaProAlaAlaAlaProAla

 541 GCTCCAGCACCTGCTGCACCGGCCGCCCCGGCGCCCGCTGCCCCCGCAGCAGCCCCAGCC 600
     AlaProAlaProAlaAlaProAlaAlaProAlaProAlaAlaProAlaAlaAlaProAla

 601 GCACCCGCTCCAGCAGCTCCCGCAGCCCCAGCACCCGCAGCACCAGCCGCCGCGCCAGCG 660
     AlaProAlaProAlaAlaProAlaAlaProAlaProAlaAlaProAlaAlaAlaProAla

 661 GCCCCGGCCCCTGCCGCGCCCGCTGCTCCCGCCCCTGCTGCCCCAGCCGCCGCTCCTGCG 720
     AlaProAlaProAlaAlaProAlaAlaProAlaProAlaAlaProAlaAlaAlaProAla

 721 GCACCTGCGCCCGCCGCGCCGGCAGCGCCGGCACCGGCAGCTCCGGCGGCCGCGCCTGCA 780
     AlaProAlaProAlaAlaProAlaAlaProAlaProAlaAlaProAlaAlaAlaProAla

 781 GCTCCTGCACCGGCGGCTCCAGCAGCCCCGGCGCCGGCCGCACCTGCGGCGGCGCCCGCG 840
     AlaProAlaProAlaAlaProAlaAlaProAlaProAlaAlaProAlaAlaAlaProAla

 841 GCGCCTGCACCCGCAGCGCCTGCGGCACCGGCCCCAGCAGCCCCTGCCGCCGCACCGGCT 900
     AlaProAlaProAlaAlaProAlaAlaProAlaProAlaAlaProAlaAlaAlaProAla

 901 GCGCCTGCCCCAGCGGCCCCCGCTGCCCCGGCCCCGGCGGCTCCAGCCGCAGCGCCTGCC 960
     AlaProAlaProAlaAlaProAlaAlaProAlaProAlaAlaProAlaAlaAlaProAla

 961 GCCCCAGCGCCCGCAGCACCGGCGGCACCAGCTCCGGCGGCGCCGGCGGCGGCTCCGGCA 1020
     AlaProAlaProAlaAlaProAlaAlaProAlaProAlaAlaProAlaAlaAlaProAla

1021 GCTCCGGCCCCTGCTGCGCCGGCTGCGCCGGCTCCGGCGGCCCCTGCGGCGGCTCCGGCC 1080
     AlaProAlaProAlaAlaProAlaAlaProAlaProAlaAlaProAlaAlaAlaProAla

1081 GCACCTGCACCTGCCGCGCCGGCTGCTCCGGCCCCGGCTGCCCCAGCAGCGGCACCAGCA 1140
     AlaProAlaProAlaAlaProAlaAlaProAlaProAlaAlaProAlaAlaAlaProAla

1141 GCGCCTGCTCCTGCGGCGCCTGCAGCTCCGGCGCCGGCAGCCCCGGCCGCCGCACCCGCG 1200
     AlaProAlaProAlaAlaProAlaAlaProAlaProAlaAlaProAlaAlaAlaProAla

1201 GCTCCAGCCCCCGCCGCTCCAGCAGCCCCCGCGCCAGCTGCACCTGCTGCCGCTCCTGCT 1260
     AlaProAlaProAlaAlaProAlaAlaProAlaProAlaAlaProAlaAlaAlaProAla

1261 GCCCCTGCTCCCGCTGCCCCCGCCGCCCCCGCCCCAGCTGCCCCCGCTGCCGCACCTGCT 1320
     AlaProAlaProAlaAlaProAlaAlaProAlaProAlaAlaProAlaAlaAlaProAla

1321 GCCCCAGCTCCCGCTGCCCCAGCCGCGCCGGCCCCCGCAGCTCCAGCCGCGGCACCAGCT 1380
     AlaProAlaProAlaAlaProAlaAlaProAlaProAlaAlaProAlaAlaAlaProAla

1381 GCCCCAGCTCCAGCGGCGCCTGCTGCCCCGGCCCCCGCGGCACCGGCTGCCGCGCCCGCA 1440
     AlaProAlaProAlaAlaProAlaAlaProAlaProAlaAlaProAlaAlaAlaProAla
```

```
1441 GCTCCAGCGCCTGCTGCACCGGCTGCTCCGGCACCCGCCGCGCCAGCAGCTGCCCCTGCG 1500
     AlaProAlaProAlaAlaProAlaAlaProAlaProAlaAlaProAlaAlaAlaProAla

1501 GCACCAGCTCCTGCTGCCCCCGCGGCACCTGCACCCGCTGCCCCGGCGGCAGCTCCCGCC 1560
     AlaProAlaProAlaAlaProAlaAlaProAlaProAlaAlaProAlaAlaAlaProAla

1561 GCGCCAGCCCCTGCAGCTCCTGCTGCACCTGCTCCTGCCGCCCCTGCTGCTGCCCCTGCT 1620
     AlaProAlaProAlaAlaProAlaAlaProAlaProAlaAlaProAlaAlaAlaProAla

1621 GCTCCAGCCCCTGCAGCACCGGCCGCTCCAGCTCCTGCCGCTCCTGCCGCTGCGCCCGCT 1680
     AlaProAlaProAlaAlaProAlaAlaProAlaProAlaAlaProAlaAlaAlaProAla

1681 GCTCCAGCCCCAGCTGCGCCAGCAGCTCCTGCACCTGCTGCCCCTGCCGCCGCCCCTGCG 1740
     AlaProAlaProAlaAlaProAlaAlaProAlaProAlaAlaProAlaAlaAlaProAla

1741 GCTCCAGCACCTGCTGCACCGGCCGCCCCGGCGCCCGCTGCCCCCGCAGCAGCCCCAGCC 1800
     AlaProAlaProAlaAlaProAlaAlaProAlaProAlaAlaProAlaAlaAlaProAla

1801 GCACCCGCTCCAGCAGCTCCCGCAGCCCCAGCACCCGCAGCACCAGCCGCCGTGCCGATT 1860
     AlaProAlaProAlaAlaProAlaAlaProAlaProAlaAlaProAlaAlaValProIle

1861 CAGAAAGTTCAGGATGATACCAAAACCCTGATTAAAACCATTGTGACCCGCATTAACGAT 1920
     GlnLysValGlnAspAspThrLysThrLeuIleLysThrIleValThrArgIleAsnAsp

1921 ATCAGCCATACCCAGAGCGTTAGCAGCAAACAGAAAGTTACCGGTCTGGATTTTATTCCG 1980
     IleSerHisThrGlnSerValSerSerLysGlnLysValThrGlyLeuAspPheIlePro

1981 GGTCTGCATCCGATTCTGACCCTGAGCAAAATGGATCAGACCCTGGCAGTTTATCAGCAG 2040
     GlyLeuHisProIleLeuThrLeuSerLysMetAspGlnThrLeuAlaValTyrGlnGln

2041 ATTCTGACAAGCATGCCGAGCCGTAATGTTATTCAGATTAGCAATGATCTGGAAAACCTG 2100
     IleLeuThrSerMetProSerArgAsnValIleGlnIleSerAsnAspLeuGluAsnLeu

2101 CGTGATCTGCTGCATGTTCTGGCATTTAGCAAAAGCTGTCATCTGCCGCAGGCAAGCGGT 2160
     ArgAspLeuLeuHisValLeuAlaPheSerLysSerCysHisLeuProGlnAlaSerGly
                                                    W100Q mutation

2161 CTGGAAACCCTGGATAGCCTGGGTGGTGTTCTGGAAGCAAGCGGTTATAGCACCGAAGTT 2220
     LeuGluThrLeuAspSerLeuGlyGlyValLeuGluAlaSerGlyTyrSerThrGluVal

2221 GTTGCACTGAGCCGTCTGCAAGGTAGTCTGCAAGATATGCTGTGGCAGCTGGATCTGAGT 2280
     ValAlaLeuSerArgLeuGlnGlySerLeuGlnAspMetLeuTrpGlnLeuAspLeuSer

2281 CCGGGTTGTTAAGCTT 2296
     ProGlyCysEnd
```

Figure S2: Coding region for the fusion protein MP-P/A(600)-huLeptin^{W100Q} cloned on pASK37. This fusion protein was used in the PK and PD studies.

Human PASylated leptin

```
      XbaI                                    NdeI
   1 TCTAGAAATAATTTTGTTTAACTTTAAGAAGGAGATATACATATGCCACATCACCACCAT 60
                                               MetProHisHisHisHis
             Amber SapI
  61 CACCATGCCTAGAGCTCTTCTGCCGCTCCTGCTGCCCCTGCTCCCGCTGCCCCCGCCGCC 120
     HisHisAlaApaSerSerSerAlaAlaProAlaAlaProAlaProAlaAlaProAlaAla
 121 CCCGCCCCAGCTGCCCCCGCTGCCGCACCTGCTGCCCCAGCTCCCGCTGCCCCAGCCGCG 180
     ProAlaProAlaAlaProAlaAlaAlaProAlaAlaProAlaProAlaAlaProAlaAla
 181 CCGGCCCCCGCAGCTCCAGCCGCGGCACCAGCTGCCCCAGCTCCAGCGGCGCCTGCTGCC 240
     ProAlaProAlaAlaProAlaAlaAlaProAlaAlaProAlaProAlaAlaProAlaAla
 241 CCGGCCCCCGCGGCACCGGCTGCCGCGCCCGCAGCTCCAGCGCCTGCTGCACCGGCTGCT 300
     ProAlaProAlaAlaProAlaAlaAlaProAlaAlaProAlaProAlaAlaProAlaAla
 301 CCGGCACCCGCCGCGCCAGCAGCTGCCCCTGCGGCACCAGCTCCTGCTGCCCCCGCGGCA 360
     ProAlaProAlaAlaProAlaAlaAlaProAlaAlaProAlaProAlaAlaProAlaAla
 361 CCTGCACCCGCTGCCCCGGCGGCAGCTCCCGCCGCGCCAGCCCCTGCAGCTCCTGCTGCA 420
     ProAlaProAlaAlaProAlaAlaAlaProAlaAlaProAlaProAlaAlaProAlaAla
 421 CCTGCTCCTGCCGCCCCTGCTGCTGCCCCTGCTGCTCCAGCCCCTGCAGCACCGGCCGCT 480
     ProAlaProAlaAlaProAlaAlaAlaProAlaAlaProAlaProAlaAlaProAlaAla
 481 CCAGCTCCTGCCGCTCCTGCCGCTGCGCCCGCTGCTCCAGCCCCAGCTGCGCCAGCAGCT 540
     ProAlaProAlaAlaProAlaAlaAlaProAlaAlaProAlaProAlaAlaProAlaAla
 541 CCTGCACCTGCTGCCCCTGCCGCCGCCCCTGCGGCTCCAGCACCTGCTGCACCGGCCGCC 600
     ProAlaProAlaAlaProAlaAlaAlaProAlaAlaProAlaProAlaAlaProAlaAla
 601 CCGGCGCCCGCTGCCCCCGCAGCAGCCCCAGCCGCACCCGCTCCAGCAGCTCCCGCAGCC 660
     ProAlaProAlaAlaProAlaAlaAlaProAlaAlaProAlaProAlaAlaProAlaAla
 661 CCAGCACCCGCAGCACCAGCCGCCGTGCCGATTCAGAAAGTTCAGGATGATACCAAAACC 720
     ProAlaProAlaAlaProAlaAlaValProIleGlnLysValGlnAspAspThrLysThr
 721 CTGATTAAAACCATTGTGACCCGCATTAACGATATCAGCCATACCCAGAGCGTTAGCAGC 780
     LeuIleLysThrIleValThrArgIleAsnAspIleSerHisThrGlnSerValSerSer
 781 AAACAGAAAGTTACCGGTCTGGATTTTATTCCGGGTCTGCATCCGATTCTGACCCTGAGC 840
     LysGlnLysValThrGlyLeuAspPheIleProGlyLeuHisProIleLeuThrLeuSer
 841 AAAATGGATCAGACCCTGGCAGTTTATCAGCAGATTCTGACAAGCATGCCGAGCCGTAAT 900
     LysMetAspGlnThrLeuAlaValTyrGlnGlnIleLeuThrSerMetProSerArgAsn
 901 GTTATTCAGATTAGCAATGATCTGGAAAACCTGCGTGATCTGCTGCATGTTCTGGCATTT 960
     ValIleGlnIleSerAsnAspLeuGluAsnLeuArgAspLeuLeuHisValLeuAlaPhe
 961 AGCAAAAGCTGTCATCTGCCGCAGGCAAGCGGTCTGGAAACCCTGGATAGCCTGGGTGGT 1020
     SerLysSerCysHisLeuProGlnAlaSerGlyLeuGluThrLeuAspSerLeuGlyGly
1021 GTTCTGGAAGCAAGCGGTTATAGCACCGAAGTTGTTGCACTGAGCCGTCTGCAAGGTAGT 1080
     ValLeuGluAlaSerGlyTyrSerThrGluValValAlaLeuSerArgLeuGlnGlySer
                                               HindIII
1081 CTGCAAGATATGCTGTGGCAGCTGGATCTGAGTCCGGGTTGTTAAGCTT 1129
     LeuGlnAspMetLeuTrpGlnLeuAspLeuSerProGlyCysEnd
```

Figure S3: Coding region for the fusion protein His_6-Apa-P/A(200)-huLeptin^{W100Q} cloned on pSB8. This fusion protein was used for the regio-selective conjugation with sCy5.5 and sCy7 and the subsequent *in vivo* imaging studies.

Simulation: One compartment model with first-order absorption for multiple s.c. P/A(600)-huLeptin^{W100Q} injections in Lep$^{ob/ob}$ mice

```
METHOD STIFF
STARTTIME=-1                  ; (h; hours)
STOPTIME=440                  ; (h; hours)
                              ; study from t = -1 to 444 h
DT = 0.001
TOLERANCE = 0.0001
DT = 0.001
DTMIN = 0.001
DTMAX = 0.1
DTOUT = 0

; Parameter definitions
Cl600 = 2.06071               ; (ml*h^-1*kg^-1)
Cl = Cl600*Wg/1000            ; (ml*h^-1; clearance)
Dose1 = 5000                  ; (pmol; s.c. dose applied)
Dose2 = 4300                  ; (pmol; s.c. dose applied)
Dose3 = 3600                  ; (pmol; s.c. dose applied)
Dose4 = 2900                  ; (pmol; s.c. dose applied)
deltaWg =   -0.054            ; +0.004 unmod. & -0.054 PASylated
V = Wg/1.79*1.21*0.06         ; (ml; plasma volume)
ka = 0.21424                  ; h^-1; lep_per. => lep
lepWT = 0.3                   ;     (pmol*ml^-1, phys. leptin level)

; Model
d/dt(Wg)                      = deltaWg              ; (g; weight in gram)
init(Wg)                      = 50
d/dt(lep_per)                 = Input - ka*lep_per
init(lep_per)                 = 0                    ; (pmol)
d/dt(lep)                     = ka*lep_per - lep*Cl/V
init(lep)                     = 0                    ; (pmol)
clep=lep/V                                           ; (pmol*ml^-1)
Input                         =(pulse(Dose1,0,1000)
                              + pulse(Dose2,120,1000)
                              + pulse(Dose3,240,1000)
                              + pulse(Dose4,360,1000))
```

Simulation: One compartment model with first-order absorption for multiple s.c. huLeptin^{W100Q} injections in Lep$^{ob/ob}$ mice

```
METHOD STIFF
STARTTIME=-1                  ; (h; hours)
STOPTIME=440                  ; (h; hours)
                              ; study from t = -1 to 444 h
DT = 0.001
TOLERANCE = 0.0001
DT = 0.001
DTMIN = 0.001
DTMAX = 0.1
DTOUT = 0

; Parameter definitions
Cl0 = 72.4                    ; (ml*h-1*kg-1)
Cl = Cl0*Wg/1000              ; (ml*h-1; clearance)
Dose1 = 5000                  ; (pmol; s.c. dose applied)
Dose2 = 5050                  ; (pmol; s.c. dose applied)
Dose3 = 5100                  ; (pmol; s.c. dose applied)
Dose4 = 5150                  ; (pmol; s.c. dose applied)
deltaWg =   0.004             ; +0.004 unmod. & -0.054 PASylated
V = Wg/1.79*1.21*0.06         ; (ml; plasma volume)
ka = 0.21424                  ; h-1; lep_per. => lep
lepWT = 0.3                   ;     (pmol*ml-1, phys. leptin level)

; Model
d/dt(Wg)                      = deltaWg               ; (g; weight in gram)
init(Wg)                      = 50
d/dt(lep_per)                 = Input - ka*lep_per
init(lep_per)                 = 0                     ; (pmol)
d/dt(lep)                     = ka*lep_per - lep*Cl/V
init(lep)                     = 0                     ; (pmol)
clep=lep/V                                            ; (pmol*ml-1)
Input                         =(pulse(Dose1,0,1000)
                              + pulse(Dose2,120,1000)
                              + pulse(Dose3,240,1000)
                              + pulse(Dose4,360,1000))
```

Curve fitting: One compartment model with first-order absorption for single s.c. P/A(600)-huLeptin^{W100Q} injections in C57BL/6 mice

```
METHOD STIFF
STARTTIME=-1                ; (h; hours)
STOPTIME=60                 ; (h; hours)
                            ; study from -1 h to 60 h
DT = 0.001
TOERANCE = 0.0001
DT = 0.001
DTMIN = 0.001
DTMAX = 0.1
DTOUT = 0

; Parameter definitions
Dose = 6888                 ; (pmol)
Cl600 = 1.3                 ; (ml*h-1*kg-1)
Cl = Cl600*Wg/1000          ; (ml*h-1; clearance)
Wg = 24                     ; (g; average weight in gram)
V = Wg*0.06                 ; (ml; plasma volume)
ka = 0.2                    ; (h-1; lep_per. => lep)

; Model
d/dt(lep_per)               = Input - ka*lep_per
init(lep_per)               = 0                          ; (pmol)
d/dt(lep)                   = ka*lep_per - lep*Cl/V
init(lep)                   = 0                          ; (pmol)
clep=lep/V                                               ; (pmol*ml-1)
Input                       = (pulse(Dose,0,1000))
```

Figure S4: Code used in Berkeley Madonna software for PK simulation and for curve fitting of the experimental PK data to obtain the absorption rate, k_a.

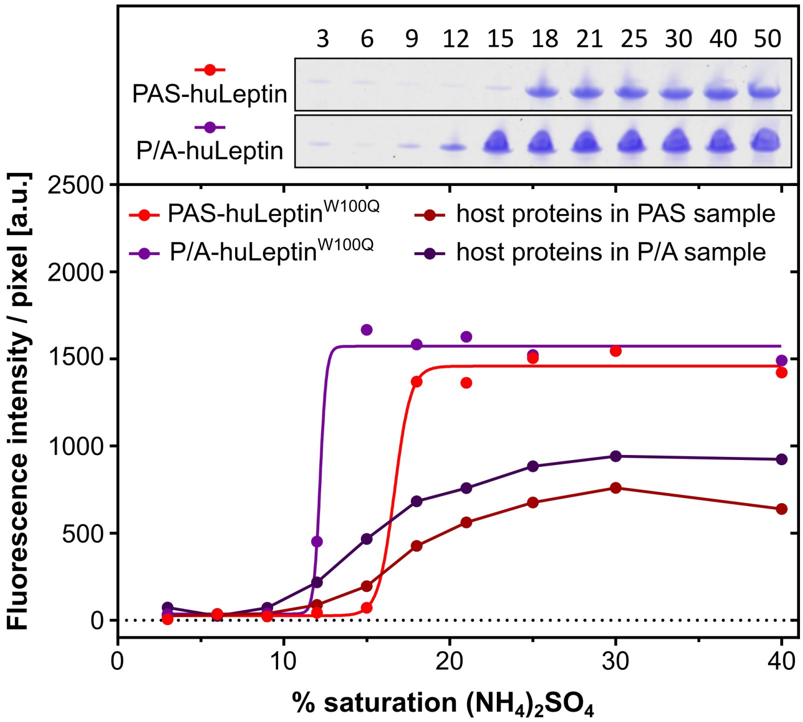

Figure S5: Purification of PASylated leptin from an *E. coli* whole cell extract using $(NH_4)_2SO_4$ precipitation. PASylated leptin was produced in the cytoplasm of *E. coli* Origami B in a soluble state. The strongly hydrophilic properties of the PAS tag, which prevent inclusion body formation, also allow the effective purification of the fusion protein by "salting out" via ammonium sulfate precipitation. Fractionated $(NH_4)_2SO_4$ precipitation, in steps of 3% saturating concentration, is shown for PAS(600)-huLeptin^{W100Q} and P/A(600)-huLeptin^{W100Q}. Samples were analyzed by SDS-PAGE (top) and the fluorescence of the Coomassie-stain was quantified using the intensity of the pixels at the center of each band.

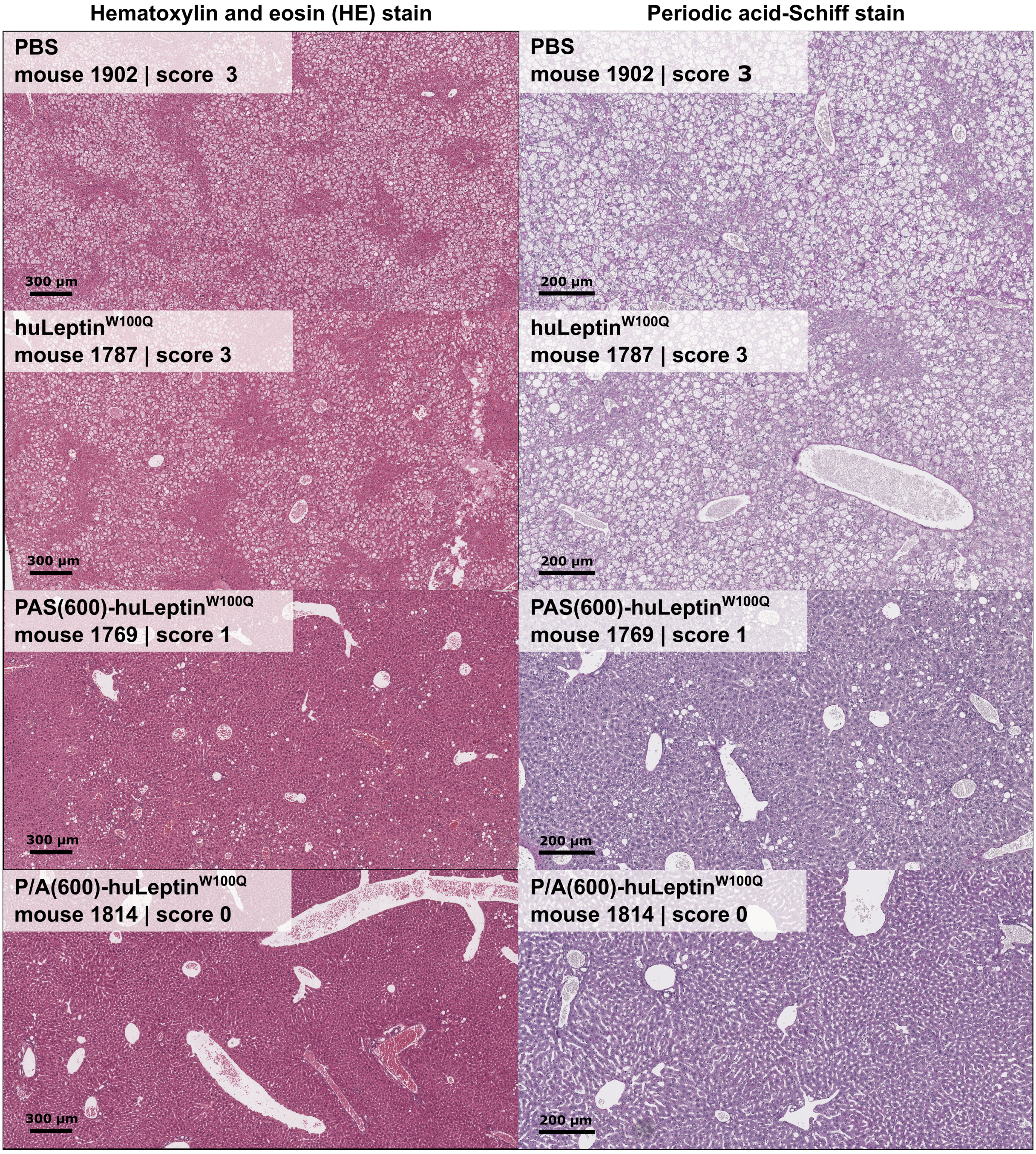

Figure S6: Histological analysis of liver tissue from leptin-treated *Lep*$^{ob/ob}$ mice by hematoxylin & eosin staining and periodate-Schiff reaction. *Lep*$^{ob/ob}$ mice from the PD study were sacrificed and liver tissue was fixed and dehydrated. 2 µm sections were prepared and stained by H&E and periodate-Schiff reaction. From each treatment cohort, a representative sample is shown for both stains along with the histology score that was determined in a blinded fashion by an expert pathologist, ranging from 0 (normal) to 3 (severe abnormalities).

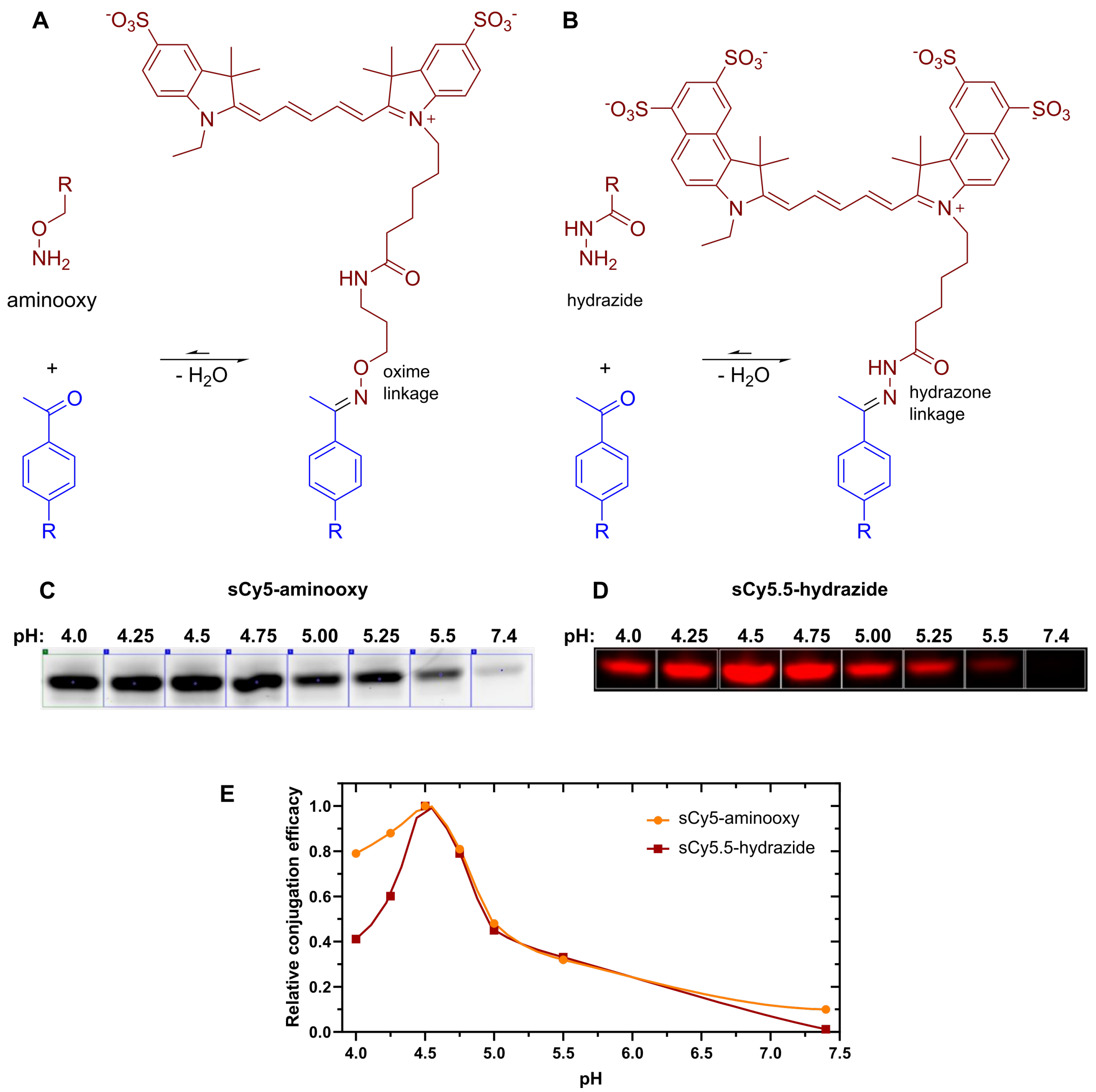

Figure S7: Conjugation of ketone-reactive dyes to the non-canonical Apa side chain in His$_6$-Apa-P/A(200)-huLeptin^{W100Q}. The condensation reaction of the aminooxy-functionalized sCy5 dye with the keto group of Apa leads to an oxime linkage (**A**), whereas the hydrazide-functionalized sCy5.5 dye results in a hydrazone (**B**). (**C–E**) Analytical conjugation reactions to investigate the influence of pH on the coupling yield. 20 µM purified His$_6$-Apa-P/A(200)-huLeptin^{W100Q} was dialyzed against PBS or 150 mM NaCl, 50 mM Na-acetate at different pH values (4–5.5) and subjected to conjugation reactions at 1:1 stoichiometric ratio with aminooxy-sCy5 (**C**) or hydrazide-sCy5.5 (**D**). The mixtures were incubated for 24 h at 4 °C, then diluted 1:10 in water and separated by SDS-PAGE under non-reducing conditions. Fluorescence intensities of the protein bands were quantified for sCy5 using an Ettan DIGE scanner in the sCy5 channel or for sCy5.5 using an Odyssey Classic LI-COR scanner in the 700 nm channel. The measured reaction products were plotted against pH (excluding the samples at pH 5.25).

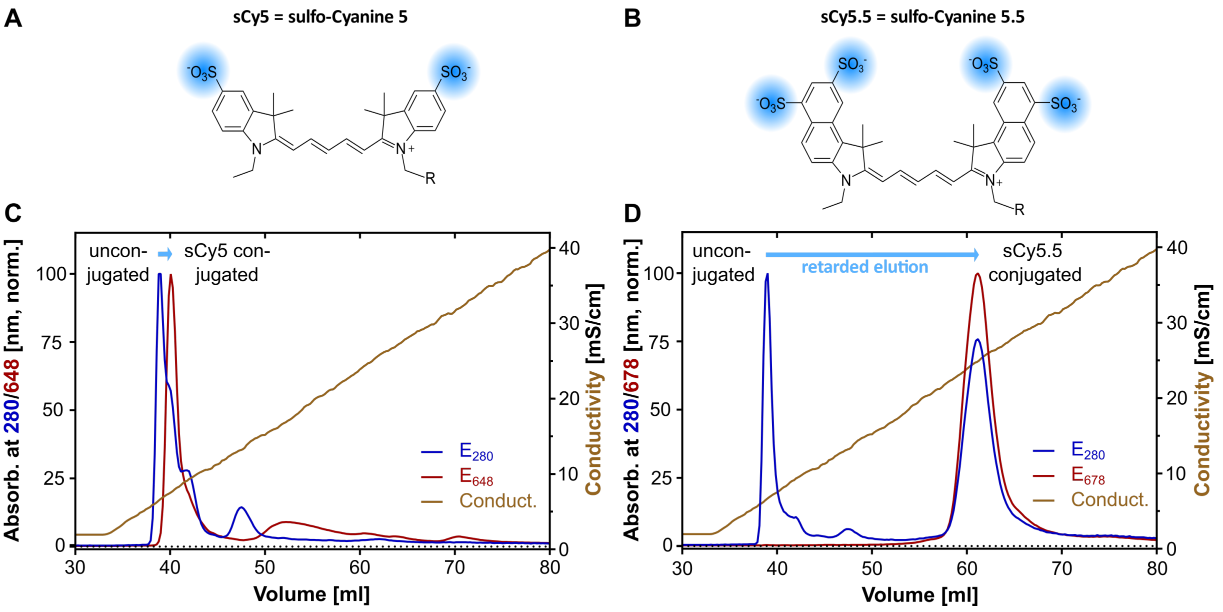

Figure S8: Isolation of the keto-conjugated PASylated leptin by AEX, taking advantage of the sulfonate groups. His_6-Apa-P/A(200)-huLeptin^{W100Q} was conjugated with aminooxy-sCy5 (**A**) or hydrazide-sCy5.5 (**B**) as described in Fig. S7. After dialysis against 10 mM Tris/HCl pH 8.5 the conjugates were separated by AEX using a Resource Q column. The two sulfonate groups in sCy5 only slightly retard the conjugate via interaction with the quaternary ammonium groups of the stationary phase (**C**). In contrast, the four sulfonate groups of the sCy5.5 dye lead to much stronger retention on the column, thus allowing the isolation of the conjugate in a separate peak (**D**).

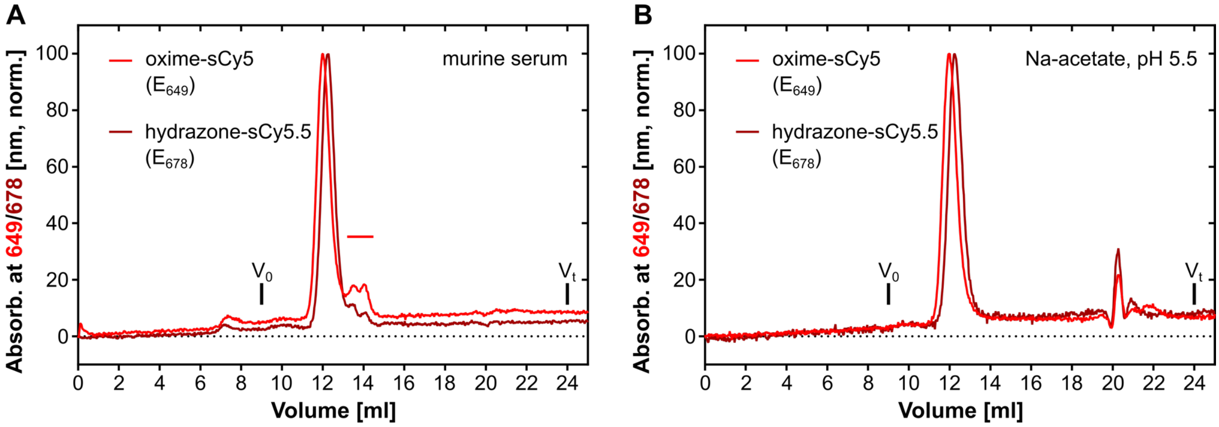

Figure S9: Stability assessment of the keto-conjugated fluorescent PASylated leptin. Purified His$_6$-Apa-P/A(200)-huLeptin^{W100Q}, conjugated either with aminooxy-sCy5 or with hydrazide-sCy5.5, was incubated 1:1 (v/v) with serum from C57Bl/6 mice (**A**) or with 500 mM Na-acetate pH 5.5 (**B**) for 6 h at 37 °C and subsequently separated by SEC using a Superdex 75 Tricorn column with PBS as running buffer. The absorbances of the fluorescent dyes were detected at 659 nm (sCy5-aminooxy) or 678 nm (sCy5.5-hydrazide).
